# Loss of PCAF in proximal tubular cells exacerbates renal fibrosis by promoting partial epithelial-to-mesenchymal transition

**DOI:** 10.1038/s12276-025-01533-x

**Published:** 2025-09-01

**Authors:** Hyunsik Kim, Jae-Hwan Kwon, Sun-Ho Lee, Seunghee Byun, Hyunseung Kim, Ho-Shik Kim, Soo-Yeon Park, Jung-Yoon Yoo, Ho-Geun Yoon

**Affiliations:** 1 Department of Biochemistry and Molecular Biology, Graduate School of Medical Science, Brain Korea 21 Project, Yonsei University College of Medicine, Seoul, Republic of Korea; 2 Department of Biochemistry, The Catholic University of Korea College of Medicine, Seoul, Republic of Korea; 3 Department of Biomedical Laboratory Science, Yonsei University MIRAE Campus, Wonju, Republic of Korea

**Keywords:** Cancer, Cell signalling, Cell biology, Epigenetics

## Abstract

Renal fibrosis is a consequence of chronic kidney disease, which is estimated to affect 10–14% of the global population. The molecular mechanisms in the pathogenesis of renal fibrosis are still unclear, and there is a lack of effective therapies. Here we identified decreased levels of p300/CBP-associated factor (PCAF) in kidney tissues with fibrosis and demonstrated that PCAF-specific knockout in proximal tubular cells accelerates renal fibrosis in both unilateral ureteral obstruction surgery and folic acid-induced models. Conversely, overexpressing PCAF in the kidney using adenovirus mitigated unilateral ureteral obstruction-induced renal fibrosis. Importantly, PCAF inhibits the epithelial-to-mesenchymal transition of proximal tubular cells by transcriptionally activating adherens junction genes. Moreover, we observed that TGF-β signaling induces lysosomal degradation of PCAF, suggesting that PCAF reduction is affected in the fibrotic milieu. These findings confirm that PCAF is a negative regulator of renal fibrosis and suggest that it could serve as a novel therapeutic target for patients with chronic kidney disease.

## PCAF identified as key regulator of renal fibrosis

Chronic kidney disease often leads to renal fibrosis, a condition in which kidney tissue becomes scarred. Researchers found that PCAF levels decrease in kidney cells during fibrosis, which worsens the condition. The authors generated mice lacking PCAF in specific kidney cells and found that these mice developed more severe fibrosis. They also investigated whether increasing PCAF expression could be beneficial and found that it reduced fibrosis. Researchers discovered that PCAF helps maintain cell structures called adherens junctions. When PCAF is low, these structures weaken, leading to a process called epithelial-to-mesenchymal transition, which contributes to fibrosis. They also found that a molecule called TGF-β triggers the breakdown of PCAF through a process involving lysosomes. The study concludes that PCAF acts as a protective factor against kidney fibrosis and suggests that therapies boosting PCAF could help treat chronic kidney disease in the future.

This summary was initially drafted using artificial intelligence, then revised and fact-checked by the author.

## Introduction

Renal fibrosis is a hallmark of various chronic kidney diseases (CKDs) and is associated with conditions such as chronic inflammation, diabetes, hypertension, metabolic dysfunction, hyperglycemia and cardiovascular disease^1^. Prompt diagnosis and early treatment are important for patients with fibrosis because it is traditionally considered an irreversible process. Furthermore, the prevalence of CKD continues to rise due to environmental factors, changes in dietary habits and the increasing incidence of metabolic syndrome, highlighting the urgency of understanding the mechanisms underlying renal fibrosis^2^.

In the kidney, several mechanisms have been implicated in the pathogenesis of fibrosis, including the activation of resident fibroblasts, epithelial-to-mesenchymal transition (EMT), endothelial-to-mesenchymal transition and the recruitment of fibrocytes^3^. These pathological processes are triggered by various stimuli, including secreted factors such as TGF-β, Wnt and TNF; environmental factors such as fatty acid oxidation, hypoxia and high glucose levels; and other external factors such as viruses^3–6^. During renal fibrosis progression, complex interactions occur among tubular epithelial cells, podocytes, fibroblasts, perivascular cells and immune cells, all contributing to disease development^5,7–10^. Among these, renal tubular epithelial cells, which make up a substantial portion of the kidney and play an essential role in response to renal injury, are recognized as the main contributor to the progression of fibrosis in the kidney^11^. For instance, damaged tubular epithelial cells can activate resident fibroblasts into myofibroblasts through extracellular vesicles or cytokines, thereby promoting fibrosis. Moreover, tubular epithelial cell-derived microRNA-rich exosomes have been reported to promote M1 polarization of macrophages, further exacerbating fibrosis^12^. Tubular epithelial cells also contribute to endothelial-to-mesenchymal transition through Myc-mediated metabolic reprogramming, accelerating renal fibrosis in response to signal transduction^13^.

Epigenetic changes regulate gene expression through chromatin remodeling and play crucial roles in biological processes such as cell development, growth and differentiation. Therefore, these changes are implicated in various diseases, including cancer, inflammation, fibrosis and autoimmune disorders^14–16^. p300/CBP-associated factor (PCAF), also known as lysine acetyltransferase 2B (KAT2B), is a histone acetyltransferase (HAT) that regulates gene expression through histone acetylation. PCAF has been identified as a key regulator in various diseases, including cancer, inflammation and fibrosis^17–21^. Recent studies have reported the downregulation of PCAF protein in human liver, stomach and cervical cancers, as well as in an isoproterenol-induced cardiac fibrosis animal model^18,22–24^. Furthermore, systemic deletion of PCAF has been shown to exacerbate vascular fibrosis by inhibiting regulatory T cells^21^. PCAF has also been reported to inhibit EMT, a critical process in fibrosis development, in organs such as the liver and cervix^24,25^. PCAF knockdown in vascular smooth muscle cells has been found to increase the mesenchymal marker α-SMA while reducing the expression of the antifibrotic factor MMP9^26^. Moreover, PCAF overexpression has been affirmed to inhibit the activation of AKT, a key regulator in the development of fibrosis^27^. While most studies on HAT proteins in fibrosis have suggested profibrotic functions, PCAF stands out as a potential antifibrotic factor.

EMT is a universal process observed in fibrotic diseases affecting various organs, including the heart, lungs and kidneys. EMT begins with the loss of cell polarity and cell adhesion, leading to a decrease in epithelial characteristics, such as E-cadherin and ZO-1, and an increase in mesenchymal traits, such as N-cadherin and vimentin^28,29^. Current studies have suggested that myofibroblasts derived from epithelial cells via EMT constitute approximately 5% of the total myofibroblast population in renal fibrosis, indicating that EMT contributes relatively little to the progression of fibrosis^9,30^. However, a more recent concept in fibrosis pathogenesis, known as partial EMT, proposes that epithelial cells exhibiting partial mesenchymal characteristics also play a significant role in renal fibrosis. These cells contribute to fibrosis by secreting secretomes such as Notch, Hedgehog (sHH) and Wnt, which activate profibrotic signaling pathways in surrounding cells, or by differentiating and activating interstitial cells, including fibroblasts^31–33^. Despite some ongoing debate, extensive research on EMT in various fibrotic diseases continues, and further studies are necessary to elucidate the physiological and molecular mechanisms underlying the EMT process.

In this study, we observed that PCAF levels are downregulated in the tubular epithelial cells of fibrotic kidneys, and proximal tubular cell (PTC)-specific PCAF deficiency deteriorates renal fibrosis following kidney damage. We also identified the functional and regulatory mechanisms by which PCAF influences the development of renal fibrosis. Collectively, our findings highlight the negative role of PCAF in the progression of renal fibrosis, providing valuable insights into its pathogenesis and potential therapeutic strategies.

## Materials and methods

### Animal studies

All animal experiments were approved by the Institutional Animal Care and Use Committee (IACUC) of Yonsei University College of Medicine (IACUC no. 2023-0025) and were conducted in accordance with standard operating procedures. Eight-week-old C57BL/6 male mice were obtained from ORIENT BIO. PCAF floxed mice (C57BL/6 background, Cyagen Biosciences) were crossed with γGT-1 Cre mice (C57BL/6 x SJL background, The Jackson Laboratory) to generate conventional PCAF-knockout mice in kidney PTCs. γGT-1 Cre mice were backcrossed onto a C57BL/6 background for at least five generations before experimental use. For all experiments involving knockout mice, littermate controls lacking Cre expression (PCAF^f/f^, γGT-1 Cre-negative) were used to ensure genetic consistency. Unilateral ureteral obstruction (UUO) surgery was performed under anesthesia by making a lateral incision in the abdomen and ligating the ureter, whereas the sham group underwent the same procedure without ligation. UUO-operated mice were euthanized 8 days after surgery. Folic acid (FA) was administered intraperitoneally at a dose of 250 mg/kg, dissolved in sodium bicarbonate. FA injected mice were euthanized 2 weeks after injection. Streptozotocin-unilateral nephrectomy (STZ-UNx) was induced by performing open unilateral nephrectomy under anesthesia, followed one week later by intraperitoneal injection of streptozotocin (50 mg/kg/day for 5 days), dissolved in 0.025 M sodium citrate buffer. Diabetic status was confirmed by fasting blood glucose levels exceeding 300 mg/dl. UNx-operated mice were euthanized 8 weeks after surgery. Adriamycin (ADR)-induced nephropathy was established by a single tail vein injection of ADR at a dose of 20 mg/kg, and mice were euthanized 6 weeks after injection. To induce PCAF overexpression, Ad5-PCAF (5 × 10^11^ Virus particles) was directly injected into the subcapsular space of the kidney, and viral infection was confirmed by detecting fluorescent signals in the kidney tissue. The mice were anesthetized via intraperitoneal injection of Zoletil (50 mg/ml) and Rompun (23.32 mg/ml). Control animals underwent sham surgery and vehicle treatment. All the mice were housed in specific-pathogen-free conditions with a 12-h light/dark cycle.

### Statistical analysis

All the data were analyzed using GraphPad Prism 10 (GraphPad Software) and are presented as the mean ± standard error of the mean (s.e.m.). Statistical analyses were conducted using one-way analysis of variance (ANOVA) for comparisons of more than two groups, with Mann–Whitney or Kruskal–Wallis tests used for post-hoc analysis, and Student’s *t*-test for comparisons between two groups. Statistical significance is indicated as follows: n.s., not significant, *P* > 0.5; *P ≤ 0.05; **P ≤ 0.01; ***P ≤ 0.001; ****P ≤ 0.0001. A *P* value of less than 0.05 was considered statistically significant.

Detailed information on materials and methods is available in the Supplementary Information.

## Results

### PCAF expression is decreased in PTCs during the progression of renal fibrosis

**Fig. 1:**
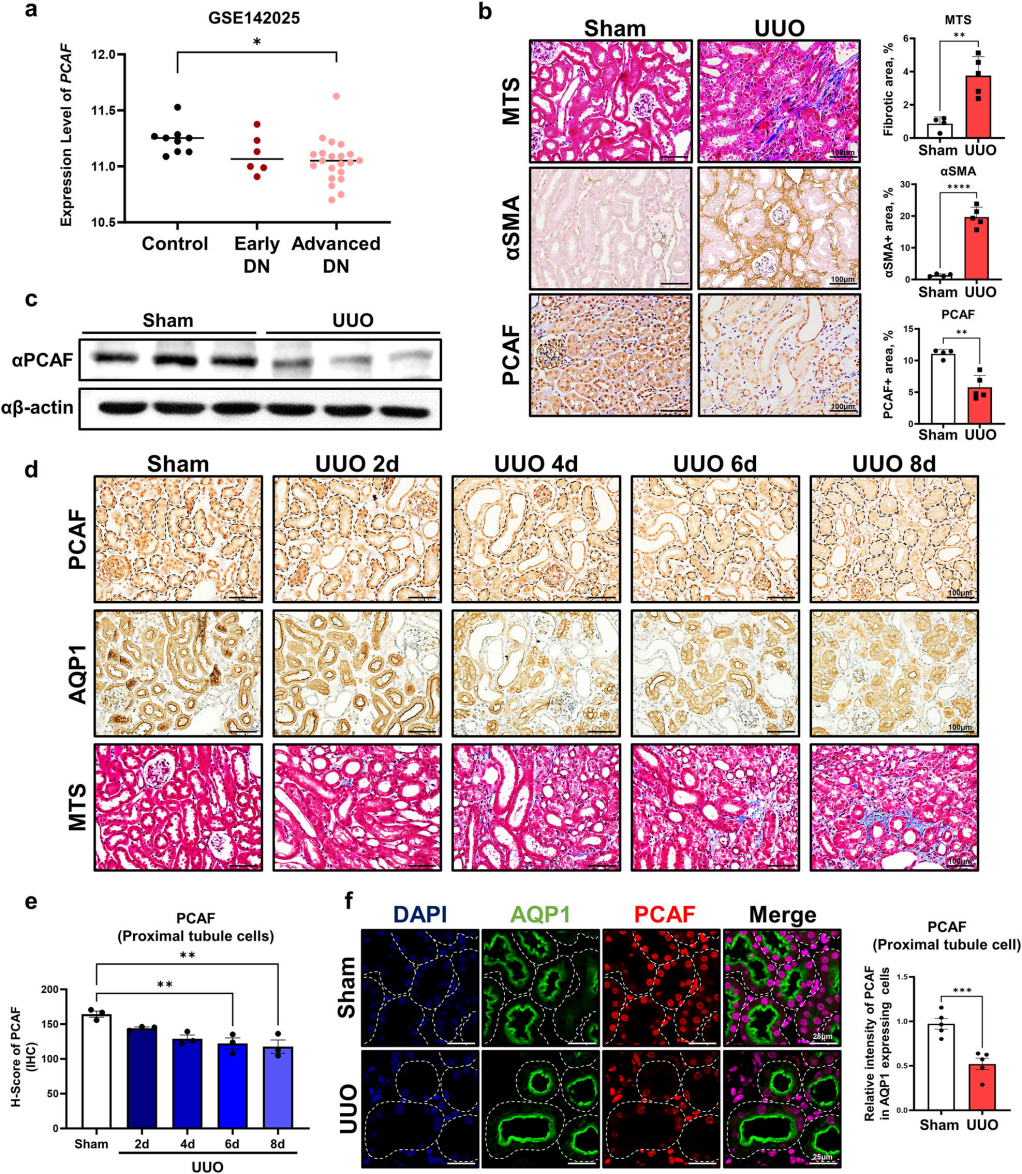
Reduction of PCAF in PTCs during the progression of renal fibrosis. **a**, mRNA levels of PCAF in the kidneys of patients with diabetes mellitus were analyzed using publicly available data, GSE142025 (early DN, *n* = 6; advanced DN, *n* = 22; control, *n* = 9). **b**, Representative images of MTS and immunohistochemistry (IHC) for α-SMA and PCAF in the kidney samples from the UUO-induced renal fibrosis model (sham, *n* = 4; UUO, *n* = 5). **c**, Protein levels of PCAF in kidney tissues from the UUO-induced renal fibrosis model. **d**, Representative IHC and MTS images showing the time-dependent PCAF expression in AQP1-positive cells in the kidney samples from the UUO-induced renal fibrosis model post-UUO surgery. The dotted lines represent individual proximal tubule units. **e**, Evaluation of PCAF expression in AQP1-positive cells using H-scoring (*n* = 3 per group). **f**, Representative images of co-immunofluorescence (IF) for PCAF and AQP1 in the kidney samples from the UUO-induced renal fibrosis model (*n* = 5 per group). The dotted lines represent individual proximal tubule units. The graph illustrates the fluorescence intensity of PCAF in AQP1-positive cells. Data are presented as mean ± s.e.m., *P < 0.05 **P < 0.01, ***P < 0.001, ****P < 0.0001 by *t*-test for two groups and ordinary one-way ANOVA for multiple groups.

To investigate the clinical relevance of PCAF in patients with CKD, we used an RNA sequencing public dataset from the kidneys of patients with diabetic nephropathy (DN), a subtype of CKD. The results confirmed that PCAF expression was significantly decreased in patients with advanced DN compared with the healthy control group (Fig. 1a). We next examined the levels of PCAF expression in several mouse models of renal fibrosis, including UUO, FA, STZ-UNx and ADR-induced renal fibrosis models. The immunohistochemistry staining showed that PCAF expression was significantly reduced in fibrotic kidneys from all four models (UUO, FA, STZ-UNx and ADR) compared with their respective controls (Fig. 1b and Supplementary Fig. 1). Consistently, reverse transcription quantitative polymerase chain reaction (RT-qPCR) and western blot analysis confirmed that PCAF mRNA and protein levels were reduced in UUO-induced fibrotic kidneys (Fig. 1c and Supplementary Fig. 2). Notably, in fibrotic kidneys, we observed a distinct decrease in PCAF expression specifically in proximal tubular epithelial cells, as demonstrated by histological staining. To further validate the reduction of PCAF in proximal tubular epithelial cells, we performed immunohistochemistry staining with AQP1, a well-known PTC marker, and other kidney parenchymal cell markers (distal tubule cell, collecting duct and glomerulus region) with PCAF on serial kidney tissue sections. Among these, PCAF expression was most significantly reduced in AQP1-positive cells, indicating that PCAF is mainly reduced in proximal tubular epithelial cells under fibrotic conditions, compared with other renal parenchymal cells (Supplementary Fig. 3). We also subsequently analyzed PCAF expression in PTCs through H-scoring in mice euthanized on days 2, 4, 6 and 8 post-UUO and found that PCAF levels were significantly decreased in proximal tubules as fibrosis exacerbated (Fig. 1d,e and Supplementary Fig. 4). Moreover, co-immunofluorescence staining of PCAF with AQP1 and other kidney cell markers in UUO-induced mouse kidney tissue further confirmed that PCAF expression was specifically reduced in AQP1-positive cells (PTCs). (Fig. 1f and Supplementary Fig. 5). These results indicate that PCAF levels are decreased in PTCs during the progression of renal fibrosis.

### PTC-specific PCAF knockout exacerbates renal fibrosis

**Fig. 2:**
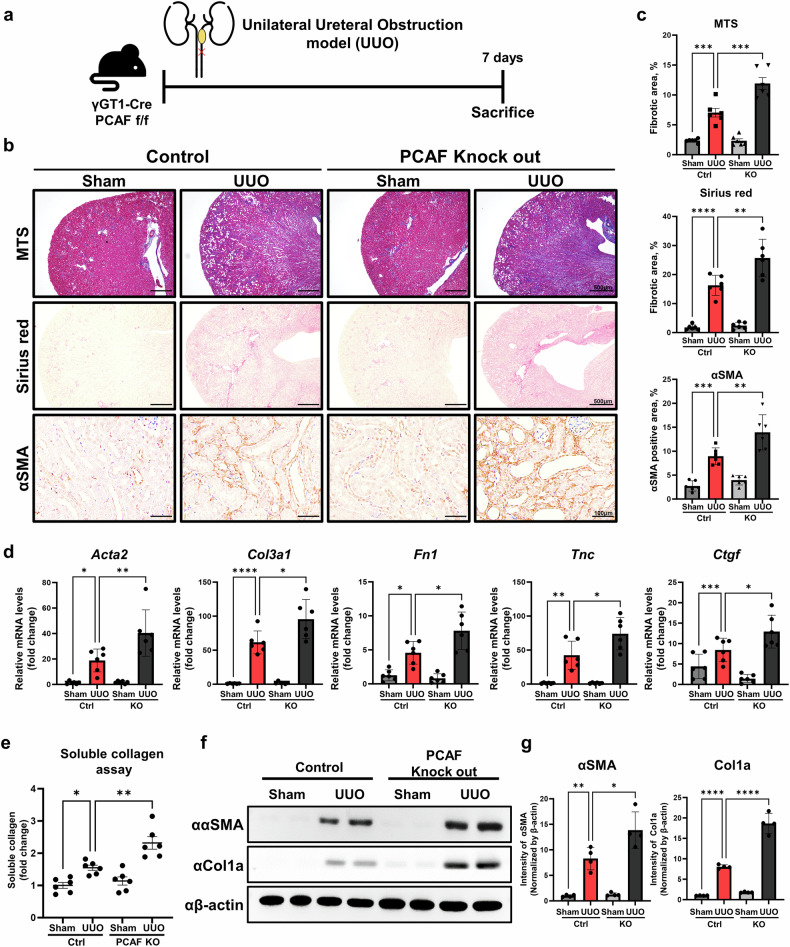
PTC-specific PCAF knockout exacerbates fibrosis progression in the UUO-induced renal fibrosis model. **a**, A schematic diagram illustrating the mouse experiment. The mice were euthanized at 7 days post-UUO surgery. **b**, Representative images of MTS, Sirius Red staining and immunohistochemistry (IHC) for α-SMA in the kidney samples from the control and PCAF-knockout UUO-induced renal fibrosis models. **c**, Quantification of the fibrotic area and the DAB-positive area of α-SMA from histological staining images (*n* = 6 per group). **d**, mRNA levels of fibrosis-related genes in kidney tissues from the control and PCAF-knockout mice in the UUO-induced renal fibrosis models (*n* = 6 per group). **e**, Soluble collagen assay performed in kidney tissues from the control and PCAF-knockout mice in the UUO-induced renal fibrosis models (*n* = 6 per group). **f**, Protein levels of fibrotic markers in kidney tissues from the control and PCAF-knockout mice in the UUO-induced renal fibrosis model. **g**, The graphs represent quantification of protein intensity levels from western blot analysis, normalized to β-actin (*n* = 6 per group). Data are presented as mean ± s.e.m., *P < 0.05 **P < 0.01, ***P < 0.001, ****P < 0.0001 by *t*-test for two groups and ordinary one-way ANOVA for multiple groups.

To further validate the functional role of PCAF in PTCs during the development of renal fibrosis, we generated PTC-specific PCAF-knockout (PTC-*Pcaf*^*d/d*^) mice using γGT-1-cre and PCAF floxed mice to explore the physiological role of PCAF in PTCs in the progression of renal fibrosis (Supplementary Fig. 6a). We confirmed PCAF knockout in PTCs by co-immunofluorescence staining for AQP1 and PCAF, as well as by western blot analysis (Supplementary Fig. 6b,c). Using the PTC-*Pcaf*^*d/d*^ mice, UUO was administered to induce renal fibrosis, and the mice were euthanized 7 days post-UUO (Fig. 2a). Kidney tissues were collected to assess fibrosis and myofibroblast activation using Masson’s trichrome staining (MTS), Sirius Red and immunohistochemical staining for α-SMA. The results showed that kidneys from the UUO-induced PTC-*Pcaf*^*d/d*^ mice exhibited significantly increased fibrosis and myofibroblast activation compared with those from the control mice (Fig. 2b,c). Collagen assays, western blotting of fibrosis-related markers (α-SMA and COL1A), and RT-qPCR analysis of fibrosis-related genes (*ACTA2*, *Col3a1*, *Fibronectin*, *TNC* and *CTGF*) revealed significant increases in collagen content and fibrosis-related marker expression in the kidneys of the UUO-induced PTC-*Pcaf*^*d/d*^ mice compared with those of the control mice (Fig. 2d–g).

**Fig. 3:**
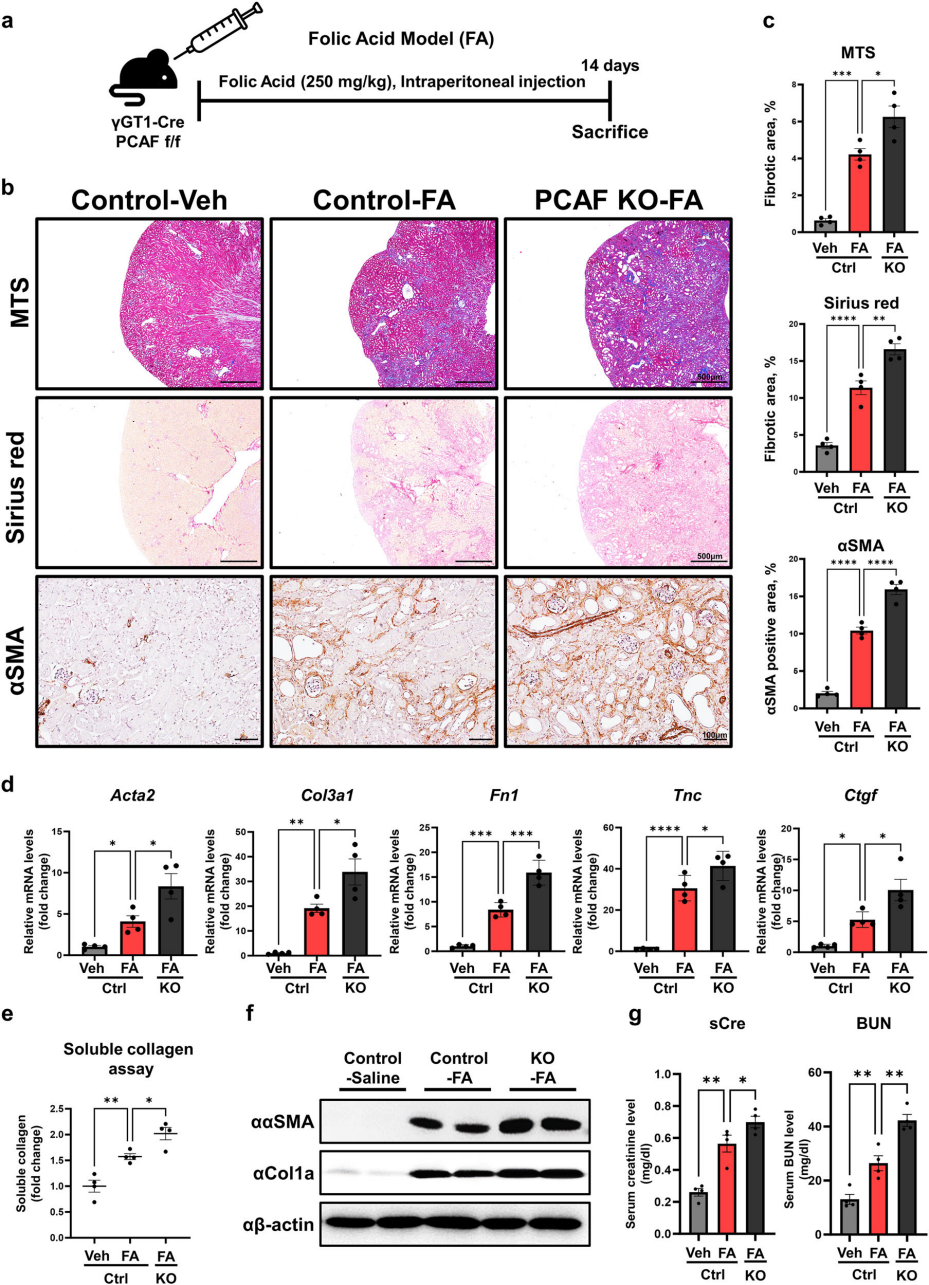
PTC-specific PCAF knockout exacerbates fibrosis progression in the FA-induced renal fibrosis model. **a**, A schematic diagram illustrating the mouse experiment. The mice were administered FA via intraperitoneal injection at a dose of 250 mg/kg and were euthanized 14 days after the injection. **b**, Representative images of MTS, Sirius Red staining and immunohistochemistry (IHC) for α-SMA in the kidney samples from the control and PCAF-knockout FA-induced renal fibrosis models. **c**, Quantification of the fibrotic area and the DAB-positive area of α-SMA from histological staining images (*n* = 4 per group). **d**, mRNA levels of fibrosis-related genes in kidney tissues from the control and PCAF-knockout mice in FA-induced renal fibrosis models (*n* = 4 per group). **e**, Soluble collagen assay performed in kidney tissues from the control and PCAF-knockout mice in FA-induced renal fibrosis models (*n* = 4 per group). **f**, Protein levels of fibrotic markers in kidney tissues from the control and PCAF-knockout mice in the FA-induced renal fibrosis model. **g**, Renal function was assessed using serum from the control and PCAF-knockout mice in the FA-induced renal fibrosis model (*n* = 4 per group). Data are presented as mean ± s.e.m., *P < 0.05 **P < 0.01, ***P < 0.001, ****P < 0.0001 by *t*-test for two groups and ordinary one-way ANOVA for multiple groups.

To further validate the negative role of PCAF in renal fibrosis progression, we used another renal fibrosis mice model using PTC-*Pcaf*^*d/d*^ mice. The FA-induced renal fibrosis model, which is caused by acute oxidative stress to PTCs, was used^34^. The PTC-*Pcaf*^*d/d*^ mice were treated with FA through intraperitoneal injection, and the mice were euthanized 2 weeks post-injection (Fig. 3a). Fibrosis progression and myofibroblast activation were significantly increased in the FA-induced PTC-*Pcaf*^*d/d*^ mice compared with the control mice, as evaluated through MTS, Sirius Red and immunohistochemistry staining for α-SMA (Fig. 3b,c). Collagen content and fibrosis-related marker expression were also significantly elevated in the kidneys of the PTC-*Pcaf*^*d/d*^ mice compared with those of the control mice (Fig. 3d–f). Moreover, kidney functional indicators, serum creatinine and blood urea nitrogen (BUN) levels were significantly elevated in the FA-induced PTC-*Pcaf*^*d/d*^ mice compared with the control mice, indicating impaired kidney function (Fig. 3g). Therefore, these results suggest that the ablation of PCAF in PTCs plays a crucial role in the progression of renal fibrosis.

### PCAF in PTCs regulates EMT through transcriptional regulation of the adherens junction protein ZO-1 during renal fibrosis

**Fig. 4:**
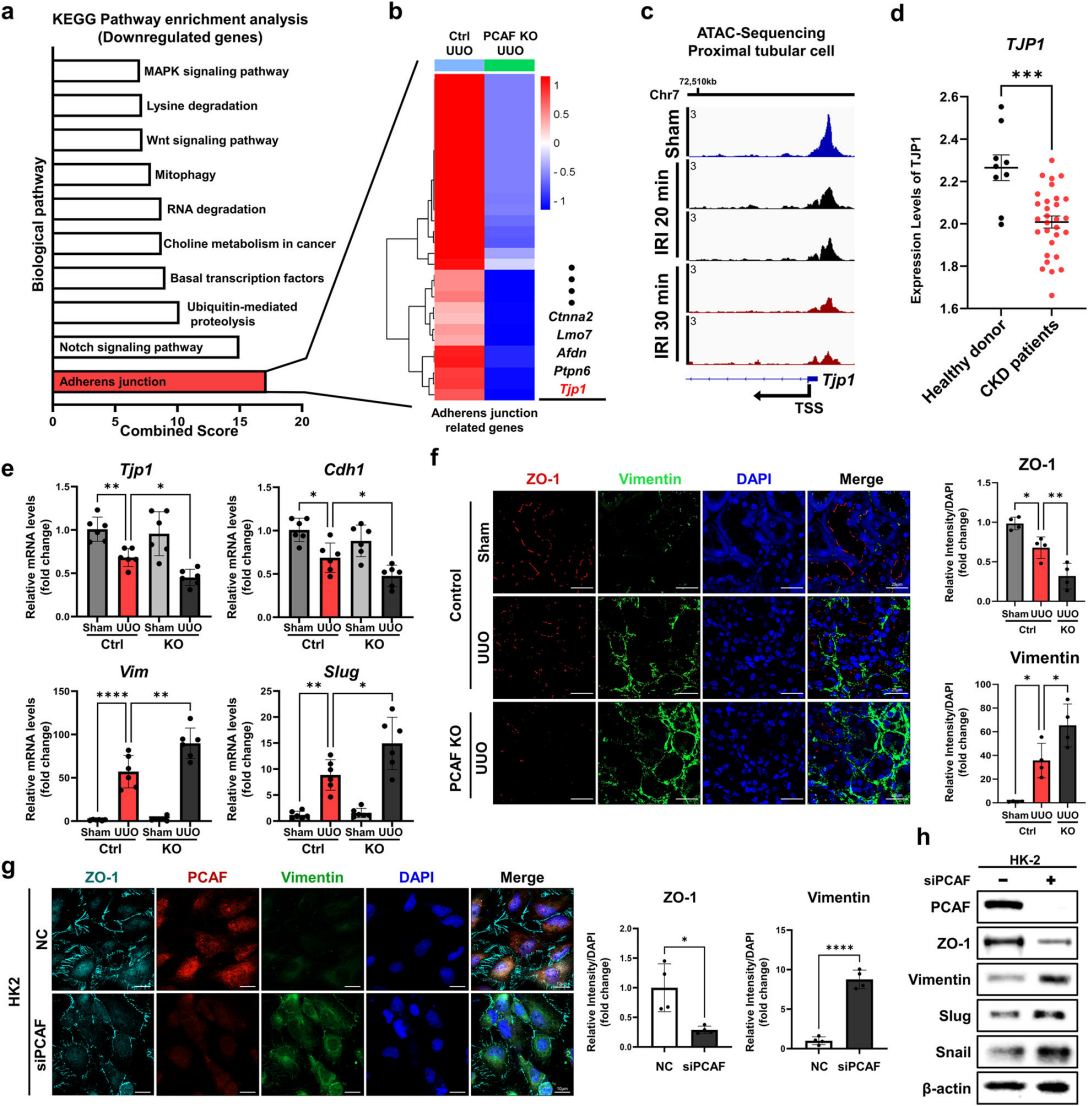
PCAF in PTCs suppresses the progression of renal fibrosis by maintaining adherens junction and inhibiting EMT. **a**, Biological pathway analysis of downregulated genes (fold change >2) in the kidneys of the PTC-specific PCAF-knockout mouse models using the KEGG database. **b**, A heatmap showing downregulated adherens junction-related genes from the KEGG database, including TJP1, in the proximal tubule-specific PCAF-knockout mice compared with the control mice after-UUO surgery. **c**, ATAC-sequencing results showing reduced chromatin accessibility at the TJP1 gene promoter in PTCs from the IRI mouse model. Public ATAC-sequencing data were obtained from GSE197814. **d**, Expression levels of *TJP1* in kidneys from patients with CKD (DN, FSGS, IgAN and lupus nephropathy) and healthy donors. **e**, mRNA levels of EMT signature genes in kidney tissues from the control and PCAF-knockout mice in the UUO-induced renal fibrosis model (*n* = 6 per group). **f**, Representative immunofluorescence images of ZO-1 and vimentin in the kidney samples from the control and PCAF-knockout mice in the UUO-induced renal fibrosis models. **g**, Representative immunofluorescence images showing changes in ZO-1 and vimentin expression upon PCAF siRNA transfection in HK2 cells. **h**, Protein levels of EMT signature markers upon PCAF siRNA transfection in HK2 cells. Data are presented as mean ± s.e.m., *P < 0.05 **P < 0.01, ***P < 0.001, ****P < 0.0001 by *t*-test for two groups and ordinary one-way ANOVA for multiple groups.

To investigate the molecular mechanism of PCAF-mediated renal fibrosis inhibition, we performed RNA sequencing on the kidney tissues of the control and PTC-*Pcaf*^*d/d*^ mice. Upregulated and downregulated genes were classified through differential expression analysis by comparing the control mice with the PTC-*Pcaf*^*d/d*^ mice post-UUO surgery. (Supplementary Fig. 7). Kyoto Encyclopedia of Genes and Genomes (KEGG) pathway analysis using the downregulated genes identified that adherens junction was most significantly regulated by PCAF depletion in PTCs (Fig. 4a). Cell adhesion plays a crucial role in maintaining cell polarity and epithelial cell characteristics homeostasis, and the loss of cell adhesion is also a hallmark of the EMT process^28,29^. Gene set enrichment analysis revealed that EMT-related gene sets were notably altered in the kidneys of the PTC-*Pcaf*^*d/d*^ mice compared with those of the control mice. We observed that the mesenchymal markers (N-cadherin and Slug) were further increased in the kidneys of the PTC-*Pcaf*^*d/d*^ mice compared with those of the control mice, whereas the epithelial marker E-cadherin was reduced (Supplementary Fig. 8). Hierarchical clustering of adherens junction Gene Ontology revealed that, among the reduced adherens junction-related-genes, Tjp1 was specifically identified as a representative key gene regulated by PCAF in PTCs to suppress EMT (Fig. 4b). Furthermore, we confirmed the presence of the reported consensus binding motif of PCAF in the promoter region of the *Tjp1* gene^35^ (Supplementary Fig. 9). Using publicly available assay for transposase-accessible chromatin (ATAC) sequencing data from renal PTCs in an ischemia–reperfusion injury (IRI) renal fibrosis mouse model, we found that chromatin accessibility decreased near the transcription start site of the *Tjp1* gene upon the progression of fibrosis (Fig. 4c). Moreover, we confirmed that the expression of *TJP1* was decreased in the kidneys of the CKD patient groups, including DN, focal segmental glomerulosclerosis, IgA nephropathy and lupus nephropathy, compared with those of healthy donors (Fig. 4d). By using public single-cell RNA sequencing data, we verified that the gene expression of *TJP1* was decreased in the kidney PTCs of the patients with CKD compared with those of the healthy donors (Supplementary Fig. 10). In both UUO and FA-induced renal fibrosis models, EMT signatures, including reduced *Tjp1* expression, were significantly increased in the kidneys of the PTC-*Pcaf*^*d/d*^ mice compared with those of the control mice (Fig. 4e and Supplementary Fig. 11). Co-immunofluorescence staining also revealed loss of ZO-1 and gain of vimentin expression, which is a mesenchymal marker, in the kidneys of the PTC-*Pcaf*^*d/d*^ mice compared with those of the control mice (Fig. 4f). To further validate the antifibrotic role of PCAF in PTCs, we examined the effect of PCAF knockdown in the human-derived PTC line, HK-2. PCAF knockdown resulted in a significant reduction in the epithelial marker ZO-1 and an increase in the mesenchymal marker vimentin compared with cells transfected with nontargeting (NC) siRNAs, as shown by co-immunofluorescence and western blot analyses (Fig. 4g,h). We also observed morphological transition to mesenchymal characteristics (spindle shape) upon PCAF knockdown in HK2 cells (Supplementary Fig. 12). These results were consistent with our in vivo data, which demonstrated that PCAF deficiency in proximal tubular epithelial cells promotes EMT by disrupting cell adhesion and epithelial homeostasis during the progression of renal fibrosis, particularly through the regulation of *Tjp1* expression.

### PCAF overexpression attenuates renal fibrosis by inhibiting EMT progression

**Fig. 5:**
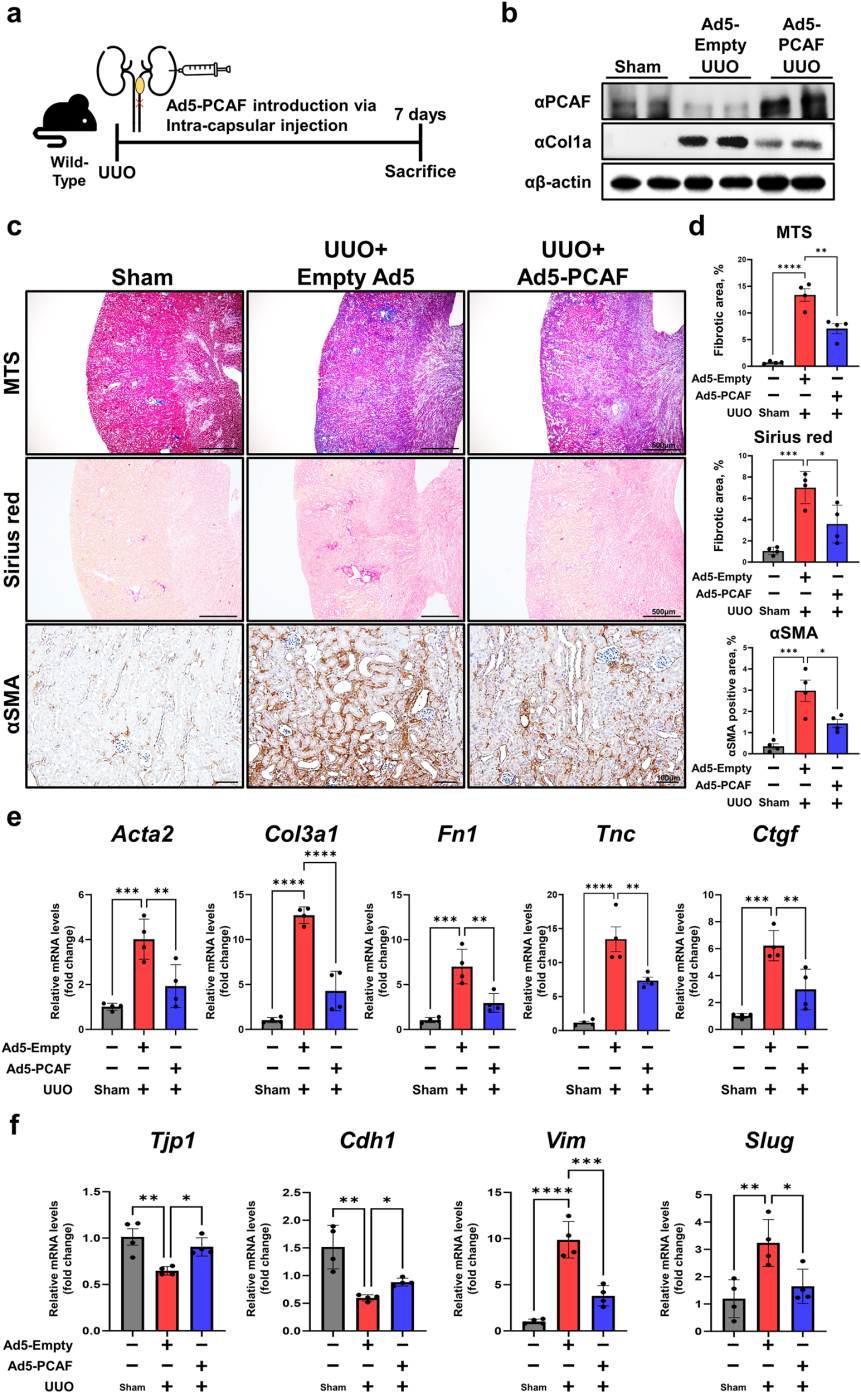
Adenovirus-mediated PCAF gene delivery attenuates renal fibrosis by inhibiting EMT progression. **a**, A schematic diagram illustrating the mouse experiment. Ad5-PCAF was administered via intracapsular injection into the kidney simultaneously with UUO surgery, and the mice were euthanized 7 days later. **b**, Protein levels of PCAF and fibrotic markers in kidney tissues from the UUO-induced renal fibrosis model with or without PCAF overexpression. **c**, Representative images of MTS, Sirius Red staining and immunohistochemistry (IHC) for α-SMA in the kidney samples from the UUO-induced renal fibrosis model with or without PCAF overexpression. **d**, Quantification of the fibrotic area and the DAB-positive area of α-SMA from histological staining images (*n* = 4 per group). **e**, mRNA levels of fibrosis-related genes in the UUO-induced renal fibrosis model with or without PCAF overexpression (*n* = 4 per group). **f**, mRNA levels of EMT signature genes in the UUO-induced renal fibrosis model with or without PCAF overexpression (*n* = 4 per group). Data are presented as mean ± s.e.m., *P < 0.05 **P < 0.01, ***P < 0.001, ****P < 0.0001 by *t*-test for two groups and ordinary one-way ANOVA for multiple groups.

Given our findings that PCAF negatively regulates renal fibrosis progression, we next tested whether overexpression of PCAF ameliorates UUO-induced renal fibrosis. To do this, we constructed an adenovirus serotype 5 (Ad5-PCAF) vector to overexpress PCAF in mouse kidney. PCAF overexpression was verified by infecting HK2 cells with Ad5-PCAF (Supplementary Fig. 13). Ad5-PCAF was injected into the intracapsule of the kidneys in UUO-induced mice, and viral delivery was confirmed by fluorescence microscopy (Fig. 5a and Supplementary Fig. 14). We examined the expression levels of PCAF and fibrotic markers in kidney tissues from the UUO-operated mice with or without Ad5-PCAF injection. In the UUO-induced mice, the expression of COL1A was decreased following PCAF overexpression (Fig. 5b). We also observed that PCAF overexpression significantly attenuated fibrosis progression and reduced myofibroblast activation in the kidneys of the UUO-induced mice compared with those treated with an empty Ad5 vector (Fig. 5c,d). Fibrosis-related marker expression was also significantly reduced in the PCAF-overexpressed kidneys compared with the UUO-induced mice treated with an empty Ad5 virus (Fig. 5e). Furthermore, PCAF overexpression suppressed EMT progression in the UUO-induced renal fibrosis model, as confirmed by RT-qPCR analysis of EMT-related genes. Furthermore, the reduction of adherens junction-related-genes caused by UUO-induced renal fibrosis was rescued by PCAF overexpression (Fig. 5f and Supplementary Fig. 15). Collectively, these results corroborate our findings that PCAF is a negative regulator of renal fibrosis by regulating EMT-related genes.

### TGF-β induces the lysosomal degradation of PCAF

**Fig. 6:**
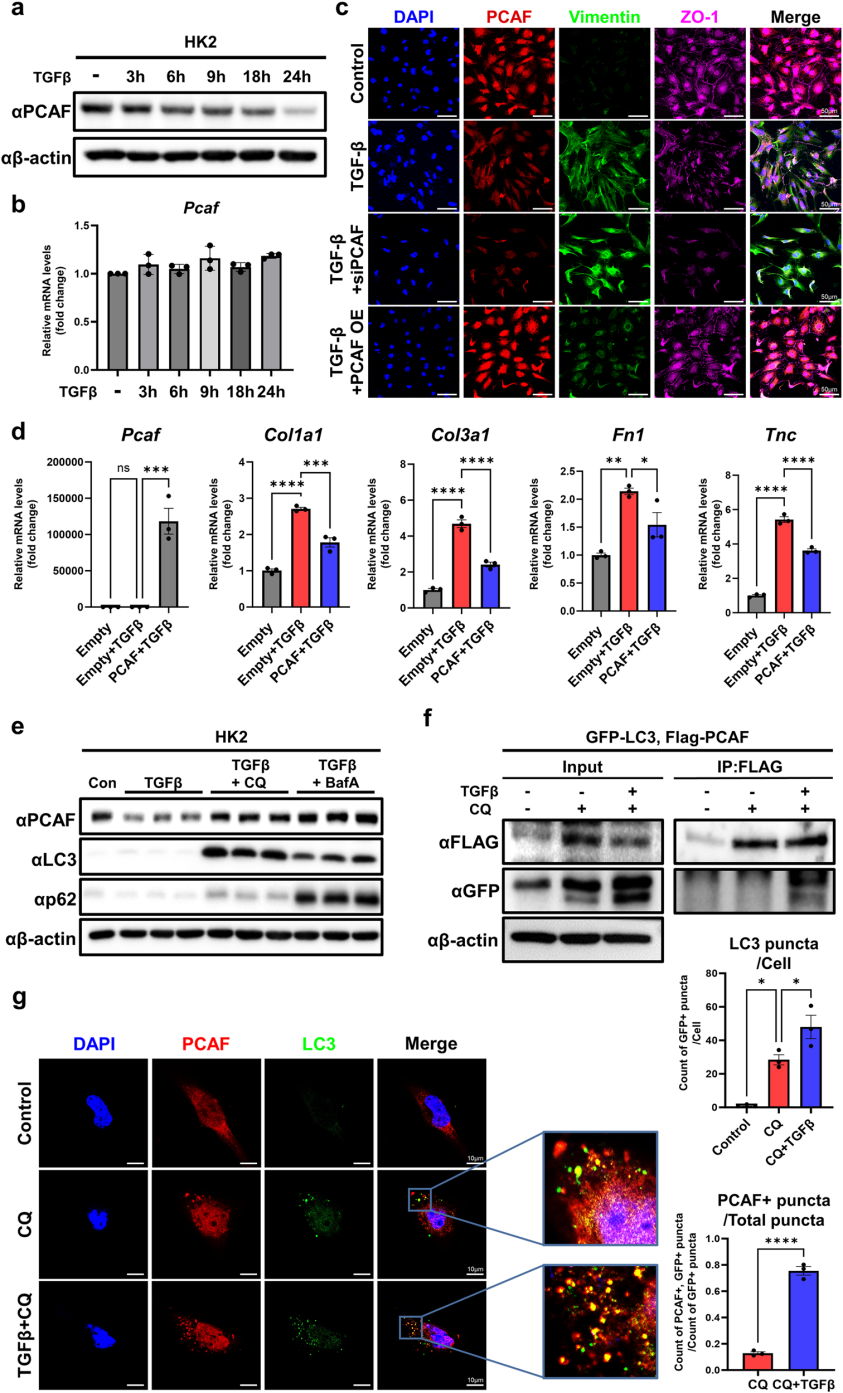
TGF-β induces PCAF degradation via the lysosomal pathway in PTCs. **a**,**b**, Time-dependent changes in PCAF protein levels (**a**) and mRNA levels (**b**) in HK2 cells after TGF-β treatment (20 ng/ml for 24 h). **c**, Representative immunofluorescence images showing changes in ZO-1 and vimentin in HK2 cells after TGF-β treatment, with PCAF knockdown or overexpression. **d**, mRNA levels of fibrosis-related genes in HK2 cells upon TGF-β treatment with PCAF overexpression. **e**, Protein levels of PCAF in HK2 cells upon TGF-β treatment with lysosomal degradation inhibitors (CQ at 100 nM and BafA at 20 nM). **f**, Immunoprecipitation of PCAF and LC3 in HK2 cells upon TGF-β treatment. FLAG-PCAF and GFP-LC3 were transfected, and CQ was added 24 h later. The cells were collected 24 h after CQ treatment. **g**, Representative immunofluorescence images showing the co-localization of PCAF and LC3 puncta in HK2 cells after TGF-β treatment. The number of LC3 puncta per cell and the number of LC3 puncta co-localized with PCAF were quantified. Data are presented as mean ± s.e.m., *P < 0.05 **P < 0.01, ***P < 0.001, ****P < 0.0001 by *t*-test for two groups and ordinary one-way ANOVA for multiple groups.

To examine the molecular mechanism how PCAF expression is reduced in PTCs during fibrosis progression, we treated the human PTC line HK2 with TGF-β, a well-known fibrogenic mediator. Western blot analysis revealed a time-dependent reduction in PCAF protein levels following TGF-β treatment, with no significant change in PCAF mRNA levels, indicating that the levels of PCAF was regulated by posttranslational modifications upon TGF-β signaling (Fig. 6a,b). It is noteworthy that other stimuli, including PDGDF, WNT3a and H_2_O_2_, significantly reduced PCAF mRNA levels in HK2 cells, indicating that alternative pathways exist in the regulation of PCAF mRNA levels in PTCs (Supplementary Fig. 16). Co-immunofluorescence data also show that TGF-β reduces the expression of PCAF and ZO-1 while increasing vimentin expression. These EMT characteristics, stimulated by TGF-β, are regulated by PCAF knockdown and overexpression (Fig. 6c). To assess the antifibrotic roles of PCAF, HK2 cells were overexpressed with PCAF and treated with TGF-β. PCAF overexpression significantly reduced the expression of fibrosis-related genes, such as *Col1a1*, *Col3a1*, *Fibronectin* and *Tnc*, which were otherwise upregulated by TGF-β treatment (Fig. 6d).

Given that PCAF levels in PTCs following TGF-β stimulation are not dependent on mRNA expression, we investigated the pathways governing PCAF protein stability in HK2 cells. We initially explored the proteasomal degradation pathway, a well-known protein degradation mechanism. MG132 and epoxomicin, proteasomal degradation inhibitors, failed to prevent the TGF-β-induced reduction of PCAF protein, suggesting that the proteasomal degradation pathway was not involved in the regulation of PCAF stability (Supplementary Fig. 17). However, lysosomal degradation pathway blockers, chloroquine (CQ) and bafilomycin A, significantly reversed the reduction of PCAF protein levels by TGF-β treatment (Fig. 6e and Supplementary Fig. 18). To further investigate whether PCAF protein stability is regulated via the lysosomal degradation pathway, we examined the interaction of LC3 to PCAF, which induces lysosomal degradation. Immunoprecipitation analysis of HK2 cells transfected with LC3 and PCAF verified that TGF-β induces the binding of LC3 to PCAF (Fig. 6f). Co-immunofluorescence staining also indicated that the co-localization of PCAF and LC3 puncta increased upon TGF-β treatment (Fig. 6g). These findings demonstrated that TGF-β signaling induces the lysosomal degradation of PCAF in PTCs, leading to EMT signature.

## Discussion

CKD accompanied by renal fibrosis is one of the most important societal problems today, significantly impairing the quality of life for many people^2^. However, molecular mechanisms underlying disease progression remain unclear due to its complex and diverse pathogenesis, and effective treatment strategies for renal fibrosis remain limited. In the present study, we investigated the role of PCAF deficiency in the progression of renal fibrosis. This work provides insights into the potential of targeting PCAF as a universally applicable therapy for patients with CKD with diverse etiologies. In contrast to other HAT proteins, such as p300 and GCN5, which are known to increase in expression during fibrosis development and mediate its progression^36–38^, our research for the first time demonstrates the PCAF-mediated antifibrosis programming during renal fibrosis progression. Our study suggests that various HAT proteins normally play physiological roles in a balanced manner, but epigenetic imbalance can cause renal fibrosis.

Several studies have reported on the protein stability of HAT proteins, such as p300, GCN5 and Tip60, and their role in modulating fibrosis. For instance, liver stiffness-induced stabilization of p300 leads to the accumulation of p300 protein and activates the differentiation of hepatic stellate cells into myofibroblasts. Similarly, during lung fibrosis development, increased p300 protein levels in alveolar type 2 cells are mediated by the deubiquitinase UCHL3^39–43^. Protein stability regulation involves the proteasomal degradation pathway and the lysosomal degradation pathway. Notably, TGF-β has been reported to induce the lysosomal degradation of proteins such as TAp63α and intracellular type 1 collagen^44,45^. There are also reports that GCN5, a KAT2 family member with high homology to PCAF, is degraded through the lysosomal pathway to promote vascular smooth muscle cell migration^42^, suggesting a possibility that PCAF stability is modulated through a similar mechanism in the context of TGF-β signaling during renal fibrosis development. Our study provides evidence that TGF-β-induced PCAF degradation occurs primarily via the lysosomal pathway rather than the proteasomal pathway. In addition to TGF-β, various signaling proteins, including TNF-α, PDGF and ET1, are known to play roles in the development of renal fibrosis^46–48^. Interestingly, in this study, we observed that, while PCAF mRNA levels did not change significantly in HK-2 cells treated with TGF-β, patient kidney tissues and fibrotic mouse models consistently showed reduced PCAF mRNA expression. To further investigate this discrepancy, we induced several fibrosis-related stimuli in HK-2 cells and found that certain stimuli, such as PDGFB and H_2_O_2_, reduced PCAF mRNA levels, suggesting that in vivo transcriptional repression of *PCAF* may be mediated by signaling pathways other than TGF-β. Thus, further studies are essential to examine whether the stability of PCAF and other HAT proteins is regulated by profibrotic factors besides TGF-β. Moreover, the kidney is composed of various cell types, and there may be differences in the expression of PCAF in each cell under renal fibrosis conditions. Therefore, it is important to understand the in vivo microenvironment during renal fibrosis and the complexity of the transcriptional regulatory mechanisms of *PCAF* in distinct renal cell types. As various cells in the kidney communicate through profibrotic factors such as TGF-β during fibrosis development, it is also necessary to understand which other kidney cells, including PTCs, are the primary recipients of profibrotic signals.

Adherens junctions are essential for cell–cell adhesion, particularly for maintaining epithelial homeostasis, and play an important role in regulating cell migration and invasion^28,29^. Furthermore, considering that the loss of adherens junctions is a prominent hallmark of EMT progression, they have recently emerged as crucial biomarkers and therapeutic targets in both cancer and fibrotic diseases^49–52^. A key component of adherens junctions, ZO-1, plays a crucial role in maintaining cell polarity by promoting strong cell–cell adhesion^53^. Recent studies have demonstrated that ZO-1 downregulation induces EMT in cancers, including liver and colon cancer, as well as in renal fibrosis^51,54–57^. Interestingly, a previous study based on chromatin immunoprecipitation sequencing in human cancer cells has revealed that PCAF regulates the transcription of cell adhesion genes, lending further support to our findings that PCAF plays a crucial role in regulating cell adhesion^35^. The kidney is composed of many epithelial cells, particularly PTCs, which make up a large portion of the kidney. Thus, understanding the role of epithelial cells in renal fibrosis development is essential, as EMT is a frequently highlighted pathogenic mechanism in renal fibrosis. Myofibroblasts, which secrete ECM during fibrosis, were initially considered to be primarily derived from fibroblasts, but some are also derived from epithelial cells, endothelial cells and others^3^. However, there is debate about the role of EMT in fibrosis, with some arguing that only a minority of myofibroblasts differentiate into fibroblasts through EMT^9,30^. Nevertheless, the expression of EMT markers has been consistently observed in fibrotic human renal biopsy tissues and renal tubular epithelial cells in mouse models of renal fibrosis^58^. Recent studies have introduced the emerging concept of partial EMT, wherein renal tubular epithelial cells retain their polarity while expressing both epithelial and mesenchymal markers during renal fibrosis pathogenesis^31,32,47,59,60^. This may play an essential role in fibrosis, not only through EMT-mediated differentiation into myofibroblasts but also through EMT-mediated changes in the pathological secretome, metabolic alterations and cell cycle arrest^31^. In the present study, we used RNA sequencing in renal proximal tubular epithelial cell-specific PCAF-knockout mouse kidneys to demonstrate that the deletion of PCAF in renal proximal tubular epithelial cells reduces adherens junctions and mediates EMT during renal fibrosis development, specifically through the regulation of ZO-1 expression. During our investigation, we observed that a partial, rather than full, EMT occurs in the proximal tubular epithelial cells during fibrosis progression. First, we found changes in EMT markers such as *Cdh1* and *Vim* in PCAF-knockout tissue from a renal fibrosis model. However, the polarity and integrity of renal tubular epithelial cells were maintained. Second, we confirmed that cell–cell junctions were preserved in TGF-β-treated or PCAF-knockdown HK2 cells. These observations suggest that even a partial EMT mediated by PCAF may be capable of significantly impacting the development of renal fibrosis. We further demonstrated that PCAF overexpression attenuated renal fibrosis with the reduction of EMT. While the present study provides new insights into the role of PCAF in EMT during renal fibrosis, further research is necessary to clarify the specific pathways through which partial EMT occurs in proximal tubular epithelial cells and how these cells communicate with surrounding cells, such as fibroblasts and endothelial cells, to promote fibrosis. Moreover, considering the pivotal role of resident fibroblasts, widely recognized as key cellular drivers of renal fibrosis, it is crucial to investigate whether PCAF also contributes to fibrotic activation in these cells. Thus, it is essential that further studies focus on the regulatory mechanisms and functional roles of PCAF in fibroblasts, along with its involvement in the interaction between epithelial cells and fibroblasts. These investigations could provide comprehensive insights into the epigenetic mechanisms underlying renal fibrosis progression and may lead to the identification of additional therapeutic targets.

Notably, a previous study reported that pharmacological inhibition of PCAF reduced renal fibrosis^61^. However, the compound used in that study, garcinol, is not selective and also inhibits other epigenetic regulators, including p300, a well-established pro-fibrotic factor, as well as HDAC11, and partially inhibits HDAC8 and SIRT3^62,63^. By contrast, our study confirmed the reduction of PCAF expression in several renal fibrosis models, particularly in PTCs. Furthermore, to investigate the specific role of PCAF in PTCs, we generated a PTC-specific PCAF-knockout mouse model and confirmed the antifibrotic function of PCAF in this context. Nevertheless, the physiological function and role of cell-specific PCAFs in the development of renal fibrosis is still poorly understood and requires further study and validation.

In summary, our study demonstrates that loss of PCAF expression during renal fibrogenesis modulates partial EMT that mediates adherens junction-related genes. We found that PCAF deficiency causes loss of adherens junction proteins, leading to the exacerbation of fibrosis by partial EMT. Finally, we elucidated that PCAF stability is regulated through the lysosomal degradation pathway in response to TGF-β signaling. Thus, our findings suggest that PCAF functions as an antifibrotic factor during the progression of renal fibrosis, offering new insights into the pathogenesis of renal fibrosis and potential therapeutic strategies.

## Supplementary information

Supplementary Information

## Data Availability

The RNA-sequencing data have been deposited in the NCBI Sequence Read Archive (SRA) (PRJNA1187834). All other data supporting the findings of this study are available upon reasonable request.
