# Supplementary material for: Loss of PCAF in proximal tubular cells exacerbates renal fibrosis by promoting partial epithelial-to-mesenchymal transition
Source: Exp Mol Med. 2025 Sep 1;57(9):2010–21. doi: 10.1038/s12276-025-01533-x (PMC12508231; doi:10.1038/s12276-025-01533-x)
Supplement: Supplementary file 1 — Supplementary Information [file 12276_2025_1533_MOESM1_ESM.pdf]

<Supplementary data for *Experimental & Molecular Medicine*>

Loss of PCAF in proximal tubular cells exacerbates renal fibrosis by promoting partial epithelial-to-mesenchymal transition

Hyunsik Kim[1#], Jae-Hwan Kwon[1#], Sun-Ho Lee[1], Seunghee Byun[1], Hyunseung Kim[1], Ho-Shik Kim[2] Soo-Yeon Park[1*], Jung-Yoon Yoo[3*], and Ho-Geun Yoon[1*]

[1]Department of Biochemistry and Molecular Biology, Severance Medical Research Institute, Brain Korea 21 PLUS Project for Medical Sciences, Yonsei University College of Medicine, Seoul 03722, Korea

[2]Department of Biochemistry, The Catholic University of Korea College of Medicine, Seoul, 06591, South Korea

[3]Department of Biomedical Laboratory Science, Yonsei University MIRAE Campus, Wonju 26493, Korea

#These authors contributed equally to this work.

*Corresponding authors

Jung-Yoon Yoo, PhD

Department of Biomedical Laboratory Science, Yonsei University MIRAE Campus, Wonju 26493, Korea. Tel: +82-33-760-2861; Fax: +82-33-760-2561; E-mail: JY_YOO@yonsei.ac.kr (J.Y.Y)

Soo-Yeon Park, PhD

Department of Biochemistry and Molecular Biology, Yonsei University College of Medicine, Seoul 120-752, Korea. Tel: +82-2-2228-0868; Fax: +82-2-312-5041; E-mail: SOOYEONP@yuhs.ac (S.Y.P)

Ho-Geun Yoon, PhD

Department of Biochemistry and Molecular Biology, Yonsei University College of Medicine, Seoul 120-752, Korea. Tel: +82-2-2228-0835; Fax: +82-2-312-5041; E-mail: YHGEUN@yuhs.ac (H.G.Y)

Supplementary material and method

Supplementary Fig. 1. PCAF expression is reduced in multiple mouse models of renal fibrosis.

Supplementary Fig. 2. PCAF mRNA expression is decreased in fibrotic kidneys of the UUO model.

Supplementary Fig. 3. PCAF expression is specifically reduced in proximal tubular cells in the UUO model.

Supplementary Fig. 4. Quantification of Masson's trichrome staining images.

Supplementary Fig. 5. Co-immunofluorescence staining of PCAF with kidney cell type–specific markers.

Supplementary Fig. 6. Generation of proximal tubular cell-specific PCAF knockout mice.

Supplementary Fig. 7. Scatter plot of DEGs between the control mice and the proximal tubular cell-specific PCAF knockout mice post-UUO surgery.

Supplementary Fig. 8. PCAF depletion in PTC promotes EMT progression.

Supplementary Fig. 9. PCAF binding motif in the promoter region of the TJP1 gene.

Supplementary Fig. 10. Single-cell RNA-sequencing data showing TJP1 expression in proximal tubular cells from CKD patients' kidneys.

Supplementary Fig. 11. Proximal tubular cell-specific PCAF knockout promotes EMT progression in the folic acid-induced renal fibrosis model.

Supplementary Fig. 12. PCAF knockdown in HK2 cells enhances EMT progression.

Supplementary Fig. 13. Confirmation of PCAF overexpression after Ad5-PCAF infection in vitro.

Supplementary Fig. 14. Confirmation of viral delivery after Ad5-PCAF injection in vivo.

Supplementary Fig. 15. PCAF overexpression restores ZO-1 expression in the UUO-induced renal fibrosis model.

Supplementary Fig. 16. mRNA levels of PCAF in HK-2 cells after treatment with fibrosis-related stimuli.

Supplementary Fig. 17. TGF-β-induced reduction of PCAF is not prevented by proteasomal degradation inhibitors in HK2 cells.

Supplementary Fig. 18. Inhibition of lysosomal degradation prevents TGF-β-induced PCAF reduction in HK2 cells

Supplementary Table 1. Primer information

Supplementary material and method

Cell culture

Human proximal tubular epithelial cells (HK2) were purchased from the American Type Culture Collection (ATCC, Manassas, VA, USA). The HK2 cells were maintained in RPMI1640 medium (Corning Inc., NY, USA) supplemented with 10% fetal bovine serum and 1% antibiotic-antimycotic solution (Corning, Manassas, VA, USA) at 37°C in a humidified atmosphere containing 5% CO_2. Prior to experimentation, HK2 cells were starved for 2 hours in serum-free medium and subsequently stimulated with 20 ng/ml TGF-β1 (Prospec, NJ, USA), 100 ng/ml PDGFB (Prospec, NJ, USA), 100 ng/ml PDGFD (Prospec, NJ, USA), 100 ng/ml FGFB (Prospec, NJ, USA), 100 ng/ml CTGF (Prospec, NJ, USA), 100 ng/ml EGF (Sigma-Aldrich, Burlington, MA, USA), 200 ng/ml Wnt3a (Prospec, NJ, USA), 10 ng/ml TNFα (Sigma-Aldrich, Burlington, MA, USA), and 500 μM H_2O_2 (Sigma-Aldrich, Burlington, MA, USA). Transient transfection of HK2 cells was performed using Lipofectamine 3000 and RNAiMAX (Thermo Fisher Scientific, Waltham, MA, USA) following the manufacturer's protocol. PCAF overexpression plasmid was cloned into pCIneo-FLAG, and siRNA for PCAF knockdown was synthesized by Genolution (Seoul, Korea). Chloroquine and bafilomycin A were purchased from Sigma-Aldrich (St. Louis, MO, USA), while epoxomicin was purchased from MedChemExpress (Monmouth Junction, NJ, USA).

Histological staining

Deparaffinized kidney tissue from the mice was rehydrated through a graded ethanol series for immunohistochemical (IHC) and immunofluorescence (IF) staining. Antigen retrieval was performed using a pressure cooker for 5 minutes using Antigen Unmasking Solution (Vector Laboratories, Burlingame, CA, USA). Following this, endogenous peroxidase activity was blocked using Dako REAL Peroxidase-Blocking Solution (Dako, Glostrup, Denmark). After antigen retrieval and peroxidase blocking, the tissue was incubated overnight at 4°C with primary antibodies against PCAF (Abcam, ab12188), AQP1 (Merck Millipore, ab2219), α-SMA (Abcam, ab5694), Zo-1 (Cell Signaling Technology, 8193p), and vimentin (Santa Cruz, sc7557). For IHC, following primary antibody incubation, the samples were treated with an HRP-conjugated anti-rabbit secondary antibody using the

Envision+ system-HRP labeled polymer anti-rabbit (Dako, Glostrup, Denmark). The signal was visualized using a 3'-diaminobenzidine (DAB) substrate (Vector Laboratories, CA, USA). For IF, the samples were treated with Dylight 488 anti-mouse and Dylight 549 anti-rabbit secondary antibodies (1:500, Vector Laboratories, CA, USA) for fluorescence labeling. After staining, the slides were mounted using a medium containing 4',6-diamidino-2-phenylindole (DAPI; Abcam, Cambridge, MA, USA) and stored at 4°C in the dark. Masson's trichrome staining was performed using the Wiegert Iron Hematoxylin Kit (Sigma-Aldrich, Burlington, MA, USA) and the MTS Kit (Sigma-Aldrich, Burlington, MA, USA) according to the manufacturer's instructions. Picro-Sirius Red staining was conducted using the Picro-Sirius Red Stain Kit (Abcam, Cambridge, UK). Quantification of stained images was performed using ImageJ, and co-localization and intensity profiles were analyzed using ZEN 3.0 (black edition) software.

Immunocytochemistry

The cells were cultured on chamber slides (SPL Life Sciences, Korea) and fixed with 4% paraformaldehyde for 15 minutes at room temperature. After fixation, the cells were washed with phosphate-buffered saline (PBS) and then permeabilized using 0.1% Triton X-100 for 30 minutes. To block non-specific binding, the cells were incubated with 3% bovine serum albumin in PBS for 30 minutes at room temperature. After blocking, the cells were incubated overnight at 4°C with primary antibodies targeting PCAF (Santa Cruz, sc13124), ZO-1 (invitrogen, 61-7300), LC3 (Santa Cruz, sc398822), and vimentin (Santa Cruz, sc7557). Following primary antibody incubation, the slides were washed with PBS and incubated with Dylight 488 anti-mouse, Dylight 549 anti-rabbit, and Dylight 649 anti-goat secondary antibodies (Vector Laboratories, Burlingame, CA, USA) for 2 hours at room temperature in the dark. After the secondary antibody incubation, the slides were washed with PBS and mounted using a mounting medium containing 4',6-diamidino-2-phenylindole (DAPI; Abcam, Cambridge, MA, USA) for nuclear staining. The slides were then stored at 4°C in the dark until analysis. Fluorescence images were captured using a Zeiss LSM780 confocal microscope (Carl Zeiss, Oberkochen, Germany), and the images were analyzed using ZEN 3.0 (black edition) software for co-localization and intensity profile analysis.

Western blot analysis

Kidney tissues and cells from the mice were lysed in lysis buffer (20 mM Tris-Cl, 150 mM NaCl, 1% Triton X-100, 1.5% MgCl2, 1 mM EDTA, 1 mM Na2VO4, 1 mM phenylmethylsulfonyl fluoride, and Xpert proteinase inhibitor cocktail (Gene Depot, TX, USA), adjusted to pH 7.5.). The lysates were briefly vortexed and then centrifuged at 12,000 rpm for 20 minutes at 4°C to collect the supernatant, which was transferred to fresh tubes. Protein concentrations were determined using a 660-nm absorbance protein assay reagent (Thermo Fisher Scientific, Waltham, MA, USA). Equal amounts of protein were mixed with SDS sample buffer (50% glycerol, 20% SDS, 0.1% bromophenol blue, 0.5% 2-mercaptoethanol) and denatured by boiling at 95°C for 5 minutes. Protein separation was conducted using SDS-polyacrylamide gel electrophoresis (SDS-PAGE), and the resolved proteins were transferred to nitrocellulose membranes (Whatman, Dassel, Germany). The membranes were blocked in Tris buffer (pH 7.4) supplemented with 0.1% (v/v) Tween-20 (Sigma-Aldrich) and 0.05 g/ml Difco skim milk (BD Biosciences, Sparks, MD, USA). Primary antibodies used encompassed PCAF (Cell signaling, 3378), aSMA (Abcam, ab5694), COL1A1 (Santa Cruz, sc59772), ZO1 (invitrogen, 61-7300), vimentin (Santa Cruz, sc7557), slug (cell signaling, 9585S), snail, LC3 (Santa Cruz, sc398822), p62 (Santa Cruz, sc28359), FLAG M2 (Sigma-Aldrich, F3165), GFP (Santa Cruz, sc9996), and β-actin (Sigma-Aldrich, A5441). After washing the membranes, they were incubated with HRP-conjugated anti-mouse (1:5000, 31430, Thermo Fisher Scientific, MA, USA) and anti-rabbit secondary antibodies (1:5000, 31460, Thermo Fisher Scientific, MA, USA). The chemiluminescent signals were detected using the Fusion SOLO S system (Vilber, Marne-la-Vallée, France), and quantitative analysis of the signals was performed using ImageJ software.

Immunoprecipitation assay

The cells were lysed in lysis buffer containing 50 mM Tris-HCl (pH 7.4), 150 mM NaCl, 0.2% Triton X-100, 0.3% NP-40, 1 mM EGTA, 1 mM EDTA, 1 mM Na3VO4, 1 mM NaF, and Xpert proteinase inhibitor cocktail (Gene Depot, TX, USA). The lysates were centrifuged at 12,000 rpm for 20 minutes at 4°C, and the supernatant was collected. A 2% input sample was set aside, and the remaining lysate

was incubated with ANTI-FLAG® M2 Affinity Gel (Millipore, A2220) at 4°C for 24 hours with gentle rotation. After incubation, the lysate and bead mixture were centrifuged at 3,000 rpm for 5 minutes at 4°C, and the supernatant was discarded. The beads were washed thrice with lysis buffer to remove unbound proteins. Bound proteins were eluted by boiling the beads in 1× SDS buffer (20% SDS, 50% glycerol, 0.1% bromophenol blue, 0.5% 2-mercaptoethanol) at 95°C for 10 minutes.

RNA isolation and quantitative RT-PCR

Total RNA isolation was performed using RNAiso Plus reagent (TaKaRa Bio, Otsu, Japan) from fresh kidney tissues and HK2 cell lines according to the manufacturer's instructions. RNA concentration and purity were measured with a NanoDrop 2000 spectrophotometer (Thermo Fisher Scientific, MA, USA). cDNA was synthesized using Cell-Script (CellSafe, Yongin, Korea) following the manufacturer's protocol, and the concentration of cDNA was normalized to α-tubulin levels. Quantitative RT-PCR (qRT-PCR) was performed using FastStart Universal SYBR Green Master (ROX) reagents (Roche, Basel, Switzerland) and an ABI Prism 7700 sequence detection system (Applied Biosystems, CA, USA). All reactions were performed in triplicate. Supplementary Table 1 lists the primer sets used for amplification.

GEO Dataset Analysis

Publicly available transcriptomic data from the GEO database (accession number: GSE142025) were analyzed. This dataset comprises kidney tissue samples obtained from patients with diabetic nephropathy (DN). According to the classification criteria defined by Ying Fan et al. (PMID: 31578193), patients were divided into early DN ($n = 6$) and advanced DN ($n = 22$) groups based on at least two independent measurements of urinary albumin-to-creatinine ratio (UACR) and estimated glomerular filtration rate (eGFR), calculated using the MDRD equation. Early DN was defined as UACR between 30 and 300 mg/g with eGFR >90 mL/min/1.73 m^2, while advanced DN was defined as UACR >300 mg/g with eGFR <90 mL/min/1.73 m^2. Control kidney samples ($n = 9$) were obtained from histologically normal regions of nephrectomy specimens from patients who underwent surgery for kidney tumors.

Adenoviral vector

The Ad5-PCAF adenovirus was generated using the Adeno-X™ Adenoviral System 3 (Takara Bio) following the manufacturer's instructions. Briefly, full-length human PCAF cDNA was subcloned into the pAdenoX-ZsGreen1 vector downstream of the CMV promoter. The recombinant plasmid was linearized and transfected into 293AD cells to produce adenovirus (Ad5). Viral particles were purified by cesium chloride (CsCl) density gradient ultracentrifugation and stored at –80°C. Transduction efficiency and infection were verified by monitoring ZsGreen1 fluorescence using fluorescence microscopy.

Soluble collagen assay

Collagen content in mouse kidney tissues was biochemically measured using the Sircol Collagen Assay Kit (Biocolor, Carrickfergus, United Kingdom) according to the manufacturer's instructions. After removing the capsules, the kidney samples (50 mg) were homogenized, and the supernatants were collected. Collagen-binding dye was then added to the samples, and they were incubated at room temperature for 1 hour, followed by centrifugation. The collagen-dye pellet was washed with ice-cold acid-salt wash reagent, dissolved in 0.5 M sodium chloride solution, and incubated at 37°C for 30 minutes. Absorbance was measured at 540 nm using a spectrophotometer to quantify collagen levels.

Kidney functional test

The blood samples were collected from the hearts of the anesthetized mice and separated into serum by centrifugation. The blood urea nitrogen (BUN) and serum creatinine levels were measured using a biochemistry analyzer (Dri-chem NX500, FUJIFILM, Tokyo, Japan).

RNA-sequencing

The total RNA was extracted from the kidney cortex samples using RNAiso Plus reagent (TaKaRa Bio, Otsu, Japan) following the manufacturer's instructions. RNA quality and concentration were measured using a NanoDrop 2000 spectrophotometer (Thermo Fisher Scientific, MA, USA). RNA sequencing

was conducted by Ebiogen (Seoul, Korea) using the QuantSeq 3' mRNA-Seq protocol (UCSC, mm10) for library preparation and sequencing. Pathway analysis was performed using PANTHER-based Gene Ontology (GO) enrichment analysis (MSigDB Hallmark) from the GO Resource (https://geneontology.org/). Visualization of heatmaps and pathway dot plots was completed using SRplot (bioinformatics.com.cn).

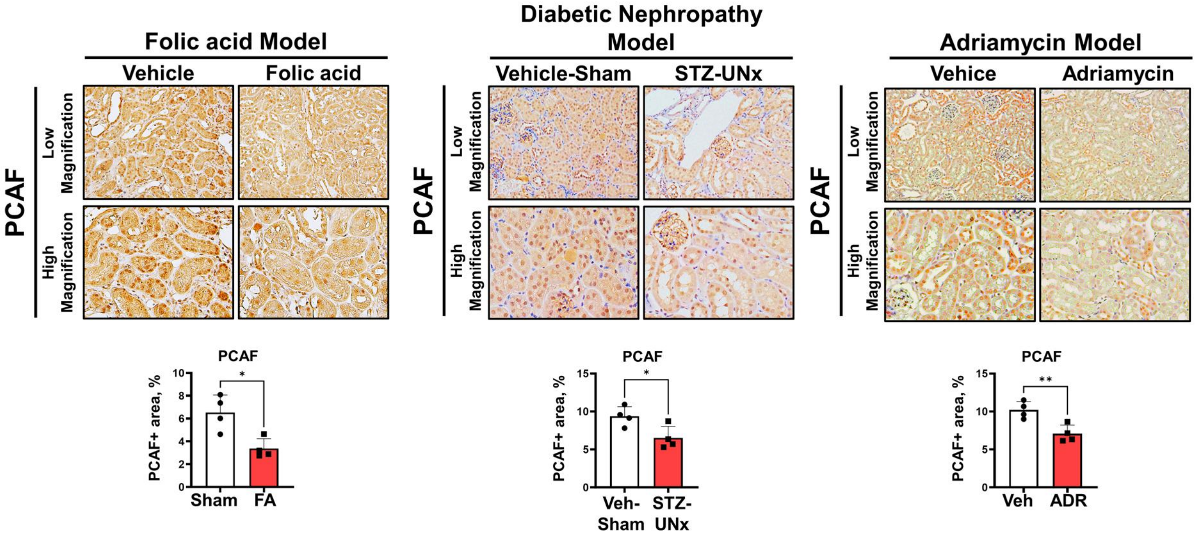

Supplementary Fig. 1. PCAF expression is reduced in multiple mouse models of renal fibrosis. Representative immunohistochemistry images of PCAF in kidney tissues from control and fibrotic mice across four different renal fibrosis models. Left to right: folic acid (FA)-induced nephropathy, adriamycin (ADR)-induced nephropathy, and uninephrectomy combined with streptozotocin (STZ+UNx)-induced diabetic nephropathy models. Quantification of the PCAF-positive area is shown below each panel (n = 4 per group). Data are presented as mean ± SEM, **P* < 0.05 and ***P* < 0.01 by unpaired t-test

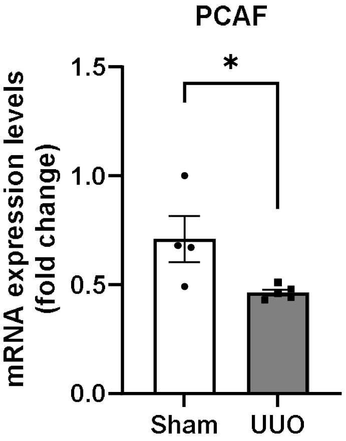

Supplementary Fig. 2. *PCAF* mRNA expression is decreased in fibrotic kidneys of the UUO model. mRNA levels of *PCAF* in kidney tissues from control and UUO-treated mice, as determined by reverse transcription-quantitative PCR (RT-qPCR) (n = 4 per group). Data are presented as mean ± SEM. **P* < 0.05 by unpaired t-test.

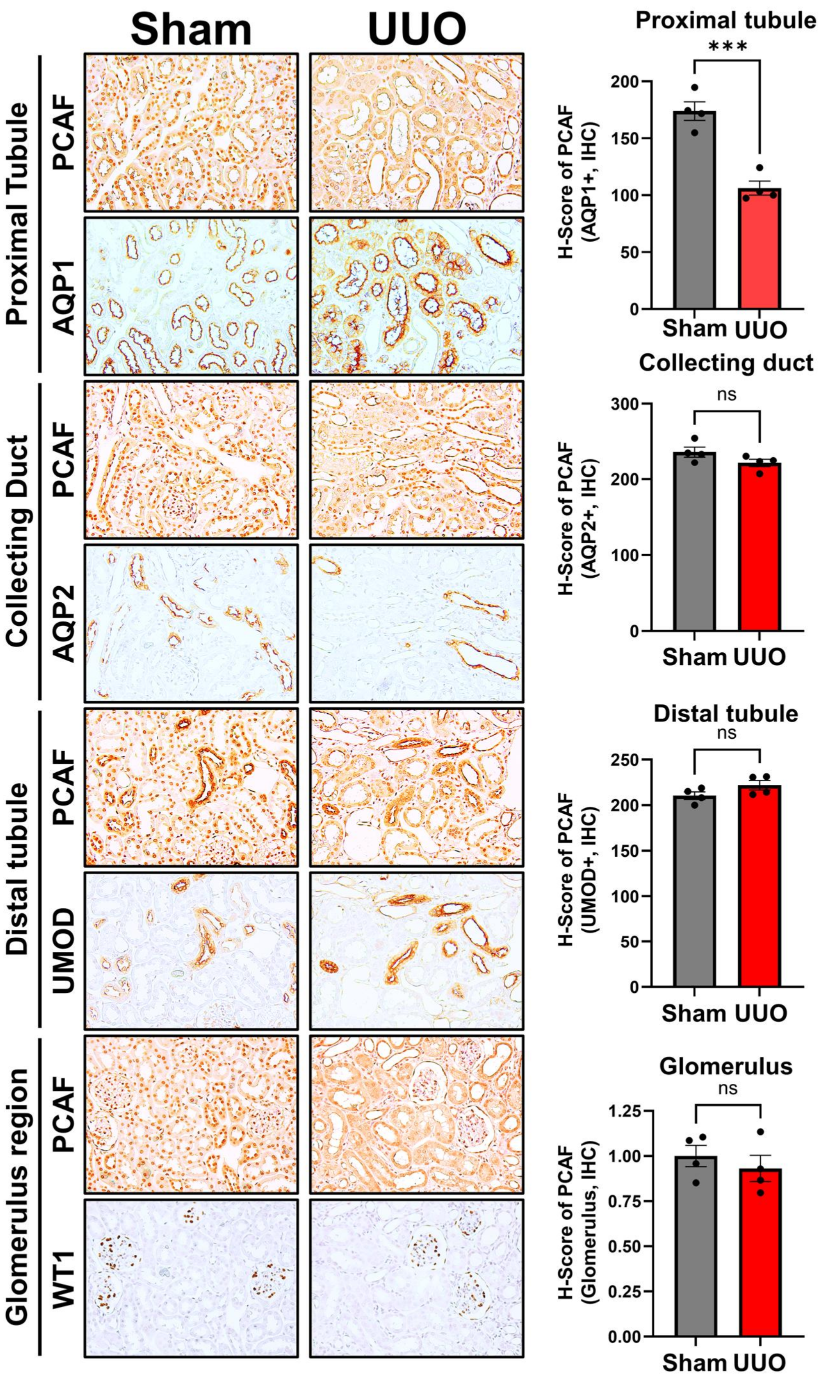

Supplementary Fig. 3. PCAF expression is specifically reduced in proximal tubular cells in the UUO model. Serial kidney sections from control and UUO-treated mice were immunohistochemically stained for PCAF and nephron–specific markers to assess PCAF expression in distinct renal cell types. AQP1 (proximal tubules), AQP2 (collecting ducts), UMOD (distal tubules), and WT1 (glomerulus) were used to define specific cell types. PCAF expression was evaluated in each specific renal cell type using H-score. Data are presented as mean ± SEM (n = 4 per group) ****P* < 0.001 by unpaired t-test.

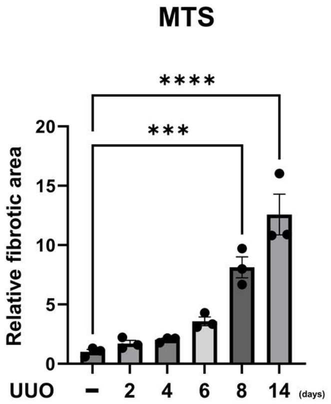

Supplementary Fig. 4. Quantification of Masson's trichrome staining images. Quantification of the fibrotic area from Masson's trichrome staining images (n = 3 per group).

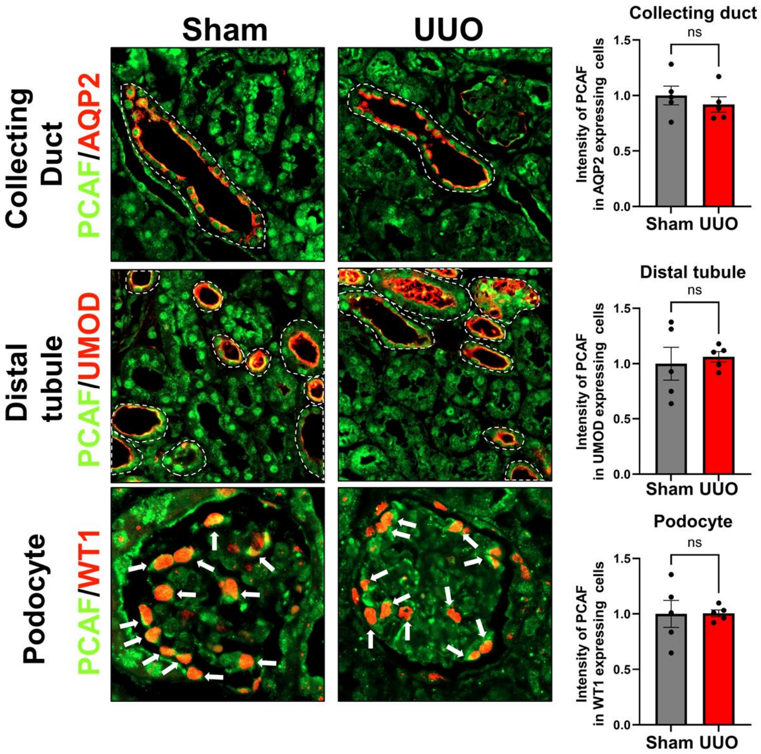

Supplementary Fig. 5. Co-immunofluorescence staining of PCAF with kidney cell type–specific markers. Representative co-immunofluorescence images of kidney tissues from UUO-induced mice, stained for PCAF (green) and cell type–specific markers (red). AQP2 was used to identify collecting duct cells, UMOD for distal tubule cells, and WT1 for podocytes. PCAF fluorescence intensity was quantified within each marker-defined cell population to assess its expression in distinct kidney cell types. Dashed lines delineate marker-positive tubular structures, and arrows indicate WT1-positive nuclei in glomeruli. Quantification of PCAF signal intensity in each cell type is shown on the right (n = 5 per group). Data are presented as mean ± SEM. ns, not significant by unpaired two-tailed t-test.

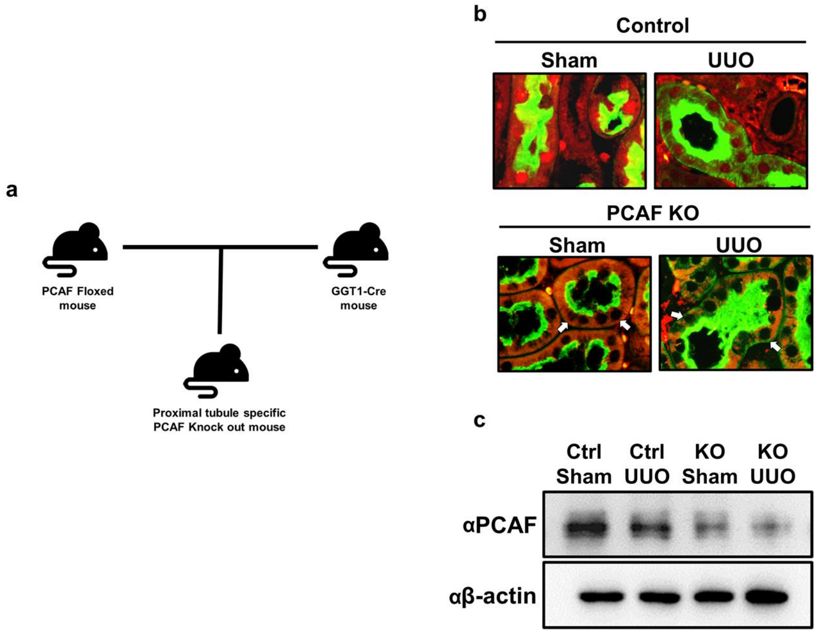

Supplementary Fig. 6. Generation of proximal tubular cell-specific PCAF knockout mice.

(a) Schematic diagram illustrating the generation of proximal tubular cell (PTC)-specific PCAF knockout mice. (b) Representative immunofluorescence images to confirm PCAF knockout in proximal tubules. AQP1 is shown in green and PCAF in red. (c) Western blot of PCAF protein levels in control and PTC-specific PCAF knockout mice with or without UUO surgery.

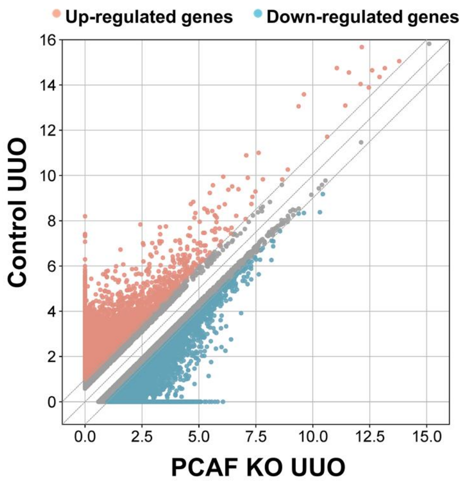

Supplementary Fig. 7. Scatter plot of DEGs between the control mice and the proximal tubular cell-specific PCAF knockout mice post-UUO surgery.

DEGs identified through the RNA sequencing of kidneys from the control and PTC-specific PCAF knockout mice post-UUO surgery.

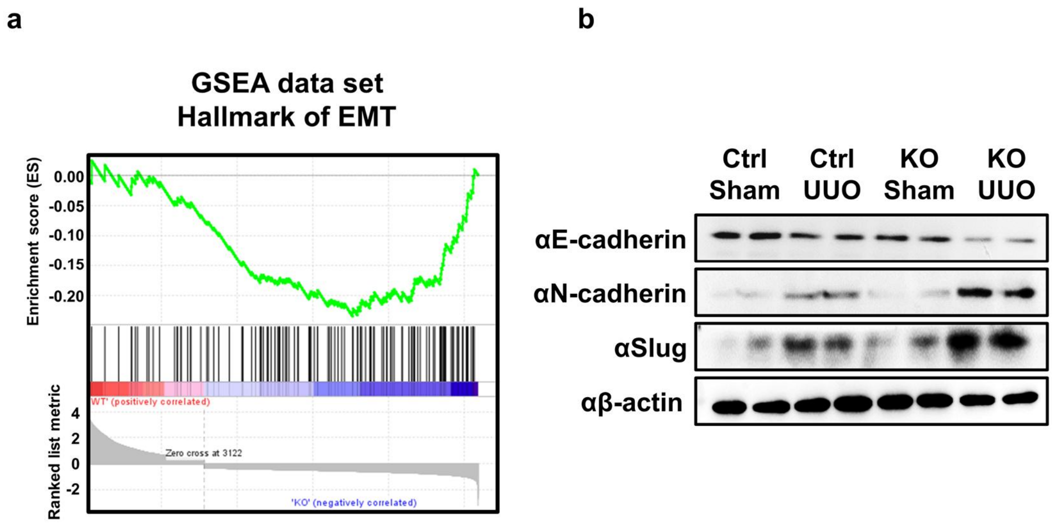

Supplementary Fig. 8. PCAF depletion in PTC promotes EMT progression.

(a) Gene set enrichment analysis (GSEA) using DEGs between the control and PTC-specific PCAF knockout mice. (b) Western blot showing protein levels of E-cadherin, N-cadherin, and slug in the control and PTC-specific PCAF knockout mice post-UUO surgery.

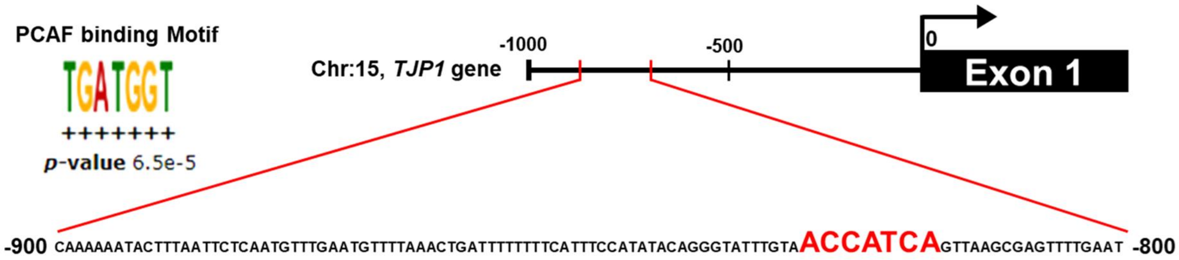

Supplementary Fig. 9. PCAF binding motif in the promoter region of the TJP1 gene.

The PCAF binding motif (TGATGGT) located at −819 bp from the transcription start site of the TJP1 gene.

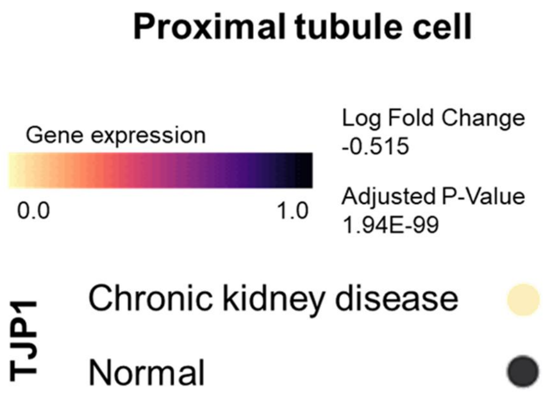

Supplementary Fig. 10. Single-cell RNA-sequencing data showing TJP1 expression in proximal tubular cells from CKD patients' kidneys.

Reduction of TJP1 expression in proximal tubular cells from CKD patients revealed by single-cell RNA-sequencing (GSE134355 and GSE183279).

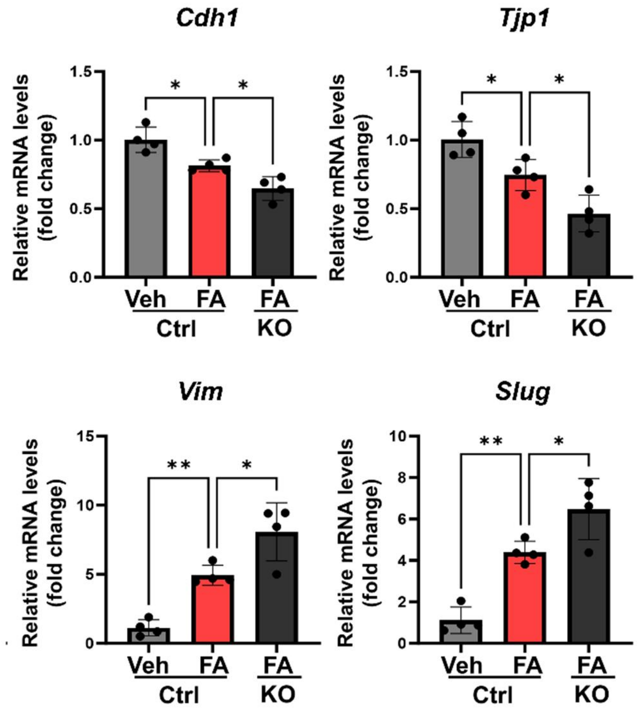

Supplementary Fig. 11. Proximal tubular cell-specific PCAF knockout promotes EMT progression in the folic acid-induced renal fibrosis model.

mRNA levels of EMT signature genes in kidney tissues from the control and PCAF knockout mice in the folic acid-induced renal fibrosis models (n = 4 per group). Data are presented as mean ± SEM, **P* < 0.05 ***P* < 0.01, ****P* < 0.001, and *****P* < 0.0001 by t-test for two groups and ordinary one-way ANOVA for multiple groups.

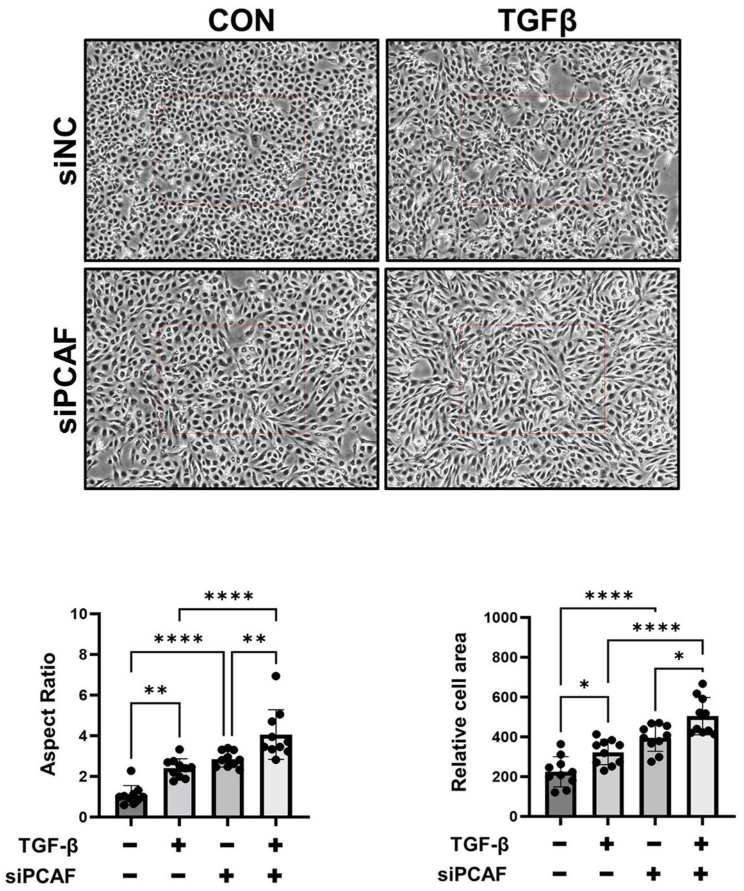

Supplementary Fig. 12. PCAF knockdown in HK2 cells enhances EMT progression.

Representative images showing cell morphology changes in PCAF knockdown HK2 cells treated with TGF-β. The graphs quantify the cell area and the aspect ratio of cell morphology. Data are presented as mean ± SEM, **P* < 0.05 ***P* < 0.01, ****P* < 0.001, and *****P* < 0.0001 by t-test for two groups and ordinary one-way ANOVA for multiple groups.

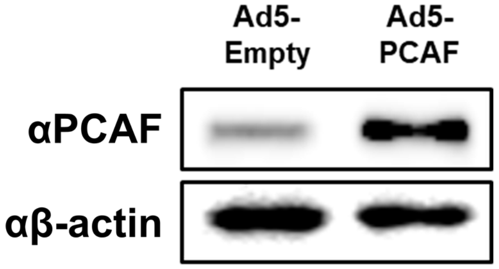

Supplementary Fig. 13. Confirmation of PCAF overexpression after Ad5-PCAF infection in vitro.

Western blot showing PCAF overexpression after Ad5-PCAF (5x10^9 VPs) infection in HK2 cells. The cells were harvested 2 days after virus injection.

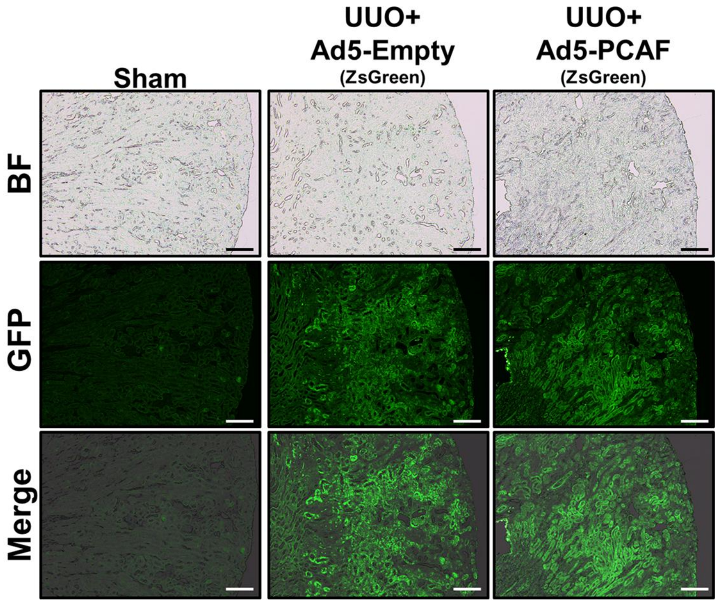

Supplementary Fig. 14. Confirmation of viral delivery after Ad5-PCAF injection in vivo.

Fluorescence detection for confirming viral infection (Ad5-PCAF) in the kidney. The mice were sacrificed 7 days after the injection, and fluorescence was observed the next day.

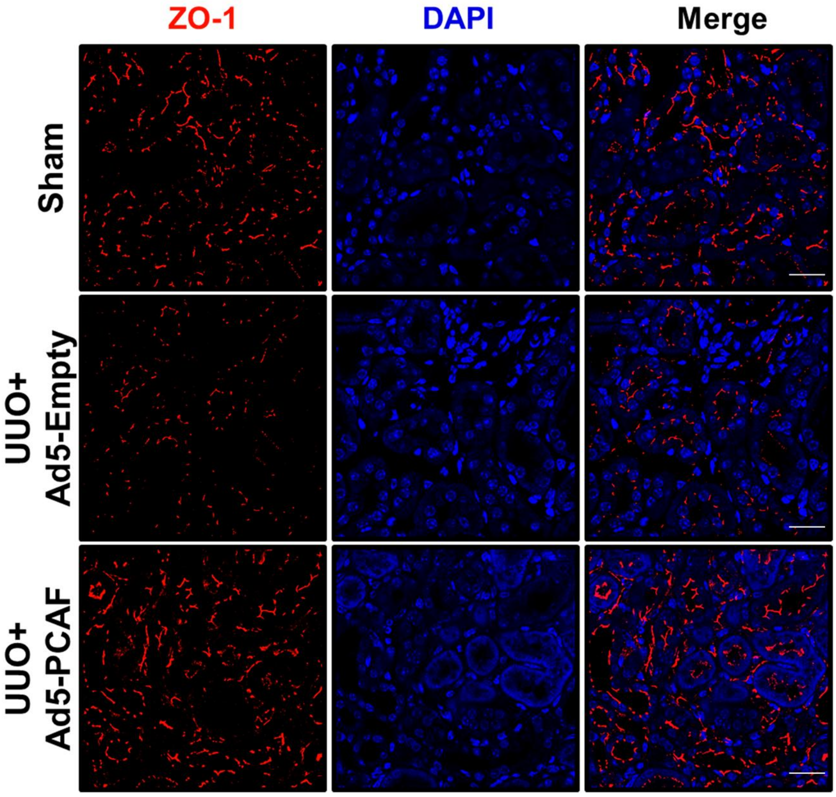

Supplementary Fig. 15. PCAF overexpression restores ZO-1 expression in the UUO-induced renal fibrosis model.

Representative immunofluorescence images showing ZO-1 expression in the UUO-induced renal fibrosis model with or without PCAF overexpression.

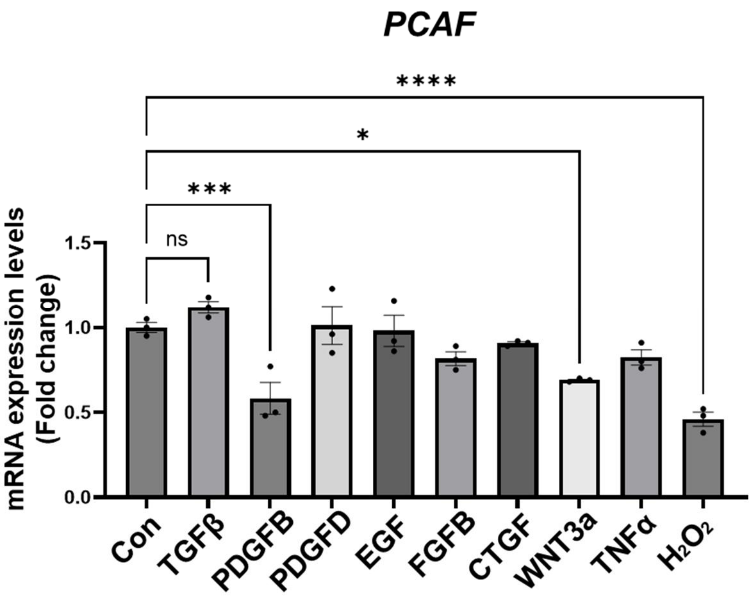

Supplementary Fig. 16. mRNA levels of *PCAF* in HK-2 cells after treatment with fibrosis-related stimuli. HK-2 cells were treated with various fibrosis-related stimuli (TGF-β, PDGFB, PDGFD, EGF, FGFB, CTGF, WNT3a, TNF α, H_2O_2). *PCAF* mRNA expression was assessed by RT-qPCR 24 hours after treatment. Data are presented as mean ± SEM, **P* < 0.05, ****P* < 0.001, and *****P* < 0.0001 by ordinary one-way ANOVA.

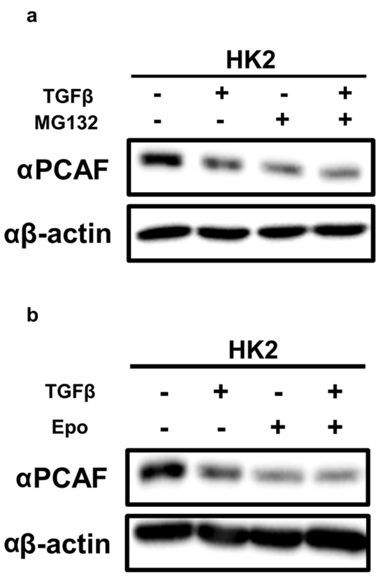

Supplementary Fig. 17. TGF-β-induced reduction of PCAF is not prevented by proteasomal degradation inhibitors in HK2 cells.

PCAF protein levels in HK2 cells treated with TGF-β and proteasomal degradation inhibitors MG132 (5 μM) and epoxomicin (5 μM).

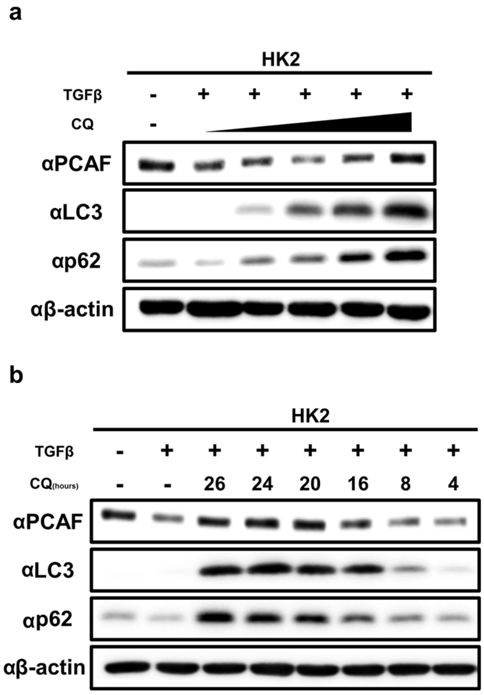

Supplementary Fig. 18. Inhibition of lysosomal degradation prevents TGF-β-induced PCAF reduction in HK2 cells

PCAF protein levels in HK2 cells treated with TGF-β and the lysosomal degradation inhibitor chloroquine in a dose-dependent manner (up to 100 nM for 24 hours) and a time-dependent manner (100 nM).

Gene		Sequence 5’-3’
Human *GAPDH*	Forward	GTCGGAGTCAACGGATTTGG
	Reverse	TGACGGTGCCATGGAATTTG
Human *PCAF*	Forward	CGAATCGCCGTGAAGAAAGC
	Reverse	CTTGCAGGCGGAGTACACT
Human *COL1A1*	Forward	TCCTGCTGGTGAGAAAGGAT
	Reverse	TCCAGCAATACCCTGAGGTC
Human *COL3A1*	Forward	TGGTCTGCAAGGAATGCCTGGA
	Reverse	TCTTTCCCTGGGACACCATCAG
Human *FN1*	Forward	AAGACCAGCAGAGGCATAAGG
	Reverse	TGTAGGGGTCAAAGCACGAG
Human *TNC*	Forward	CAGAAGCCGAACCGAAGTT
	Reverse	TTCATCAGCTGTCCAGGACAGA
Mouse *Gapdh*	Forward	GCATGGCCTTCCGTGTTCCT
	Reverse	CCCTGTTGCTGTAGCCGTATTCAT
Mouse *Pcaf*	Forward	GCCGTGTCATTGGTGGTATC
	Reverse	GGGTTCCATAGCCCTTGACT
Mouse *Acta2*	Forward	GTGACTCACAACGTGCCTATC
	Reverse	CTCGGCCAGTAGTCACGAAGG
Mouse *Col3a1*	Forward	CTAAAATTCTGCCACCCCGAA
	Reverse	AGGATCAACCCAGTATTCTCCACTC
Mouse *Fn1*	Forward	AAGACCATACCTGCCGAATG
	Reverse	GAACATGACCGATTTGGACC
Mouse *Tnc*	Forward	TGCAACGACTTCCTTTGCAC
	Reverse	GGCTCGGAGAATGACCATGT
Mouse *Ctgf*	Forward	CCACCCGAGTTACCAATGAC
	Reverse	GTGCAGCCAGAAAGCTCA
Mouse *Tjp1*	Forward	GTTGGTACGGTGCCCTGAAAGA
	Reverse	GCTGACAGGTAGGACAGACGAT
Mouse *Cdh1*	Forward	GGTCATCAGTGTGCTCACCTCT
	Reverse	GCTGTTGTGCTCAAGCCTTCAC
Mouse *Vim*	Forward	CCCTCACCTGTGAAGTGGAT
	Reverse	TCCAGCAGCTTCCTGTAGGT
Mouse *Slug*	Forward	AGATGCACATTCGAACCCAC
	Reverse	GTCTGCAGATGAGCCCTGAG

Supplementary Table 1. Primer information
